# Identification of non-invasive miRNAs biomarkers for prostate cancer by deep sequencing analysis of urinary exosomes

**DOI:** 10.1186/s12943-017-0726-4

**Published:** 2017-10-05

**Authors:** Marta Rodríguez, Cristina Bajo-Santos, Nina P. Hessvik, Susanne Lorenz, Bastian Fromm, Viktor Berge, Kirsten Sandvig, Aija Linē, Alicia Llorente

**Affiliations:** 1 Department of Molecular Cell Biology, Institute for Cancer Research, Oslo University Hospital-The Norwegian Radium Hospital, 0379 Oslo, Norway; 2 Centre for Cancer Biomedicine, Faculty of Medicine, University of Oslo, 0379 Oslo, Norway; 3 Latvian Biomedical Research and Study Centre, Riga, LV-1067 Latvia; 4 Department of Tumor Biology, Institute for Cancer Research, Oslo University Hospital-The Norwegian Radium Hospital, 0379 Oslo, Norway; 5 Department of Urology, Oslo University Hospital, 0586 Oslo, Norway; 6 Department of Biosciences, University of Oslo, 0316 Oslo, Norway

**Keywords:** Biomarkers, Exosomes, Extracellular vesicles, microRNAs, Microvesicles, noncodingRNA, Next-generation sequencing, Prostate cancer, Small RNA, Urine

## Abstract

The aim of this study was to identify microRNAs in urinary exosomes that are differently expressed in prostate cancer patients and healthy donors. For this purpose, RNA was extracted from urinary exosomes from 20 prostate cancer patients and 9 healthy males and the microRNAs were analyzed by next generation sequencing. Interestingly, 5 microRNAs – miR-196a-5p, miR-34a-5p, miR-143-3p, miR-501-3p and miR-92a-1-5p – were significantly downregulated in exosomes from prostate cancer patients. Furthermore, RT-qPCR analysis of an independent cohort of 28 prostate cancer patients and 19 healthy males confirmed that miR-196a-5p and miR-501-3p were downregulated in prostate cancer samples. These results suggest that specific microRNAs in urinary exosomes might serve as non-invasive biomarkers for prostate cancer. In particular, miR-196a-5p and miR-501-3p are promising biomarkers that need to be further studied in large patient cohorts.

**Electronic supplementary material:**

The online version of this article (10.1186/s12943-017-0726-4) contains supplementary material, which is available to authorized users.

## Background

Prostate cancer (PCa) accounts for 12% of all cancer cases worldwide, and it is the second most commonly diagnosed cancer in men [1]. There is an urgent need for early diagnosis and prognosis biomarkers for the disease [2]. Biomarkers that can be measured in biofluids, often referred to as liquid biopsies [3], avoid the discomforts and may reflect the heterogeneity of the tumor better than prostate needle biopsies. Remarkably, exosomes released by cancer cells into biological fluids contain molecules that reflect the disease status, and are considered today as a new type of liquid biopsies [4, 5]. In particular, exosomes contain microRNAs (miRNAs) [6], which are small noncoding RNAs involved in the regulation of gene expression. miRNAs have been show to play several functions in cancer, and specific miRNA signatures have been identified in several cancer types [7, 8]. Interestingly, cancer-specific miRNAs have been identified in exosomes [9], and several exosomal miRNAs may serve as PCa biomarkers [10–13]. We have here investigated the use of next generation sequencing to identify novel miRNA-based PCa biomarkers in urinary exosomes and used RT-qPCR to validate the results.

## Results

### Isolation of exosomal RNA from urine samples

Urinary exosomes were isolated by sequential centrifugation as previously described [14]. We have previously characterized these vesicles by several methods such as Western blot, electron microscopy, and mass spectrometry-based proteomics [14]. Based on these analyses we concluded that our samples are enriched in exosomes, but we cannot exclude that other extracellular vesicles co-isolate with exosomes to some extent. A material corresponding to 2.5–10 μg exosomal proteins (as measured with the BCA assay) was used for RNA isolation. Before RNA was isolated, the samples were treated with proteinase K and RNAse A to degrade potential RNA/protein complexes. Several methods have been described to isolate RNA from urinary exosomes [15], and in this study mirCury, miRNeasy and trizol were tested. After RNA isolation, the samples were treated with DNAse to degrade potential DNA. The RNA concentrations obtained were too low to be measured by Nanodrop, and therefore the samples were analyzed in Agilent RNA 6000 Pico chips. The miRNeasy Micro RNA isolation kit gave the highest RNA yield, 1–3 ng RNA from a material corresponding to 4–5 μg of exosomal protein. As shown in a typical Bioanalyzer RNA profile (Additional file 1: Figure S1), no evidence of 18S and 28S RNA was observed.

### Next generation sequencing of urinary exosomes from PCa patients and healthy controls

**Fig. 1:**
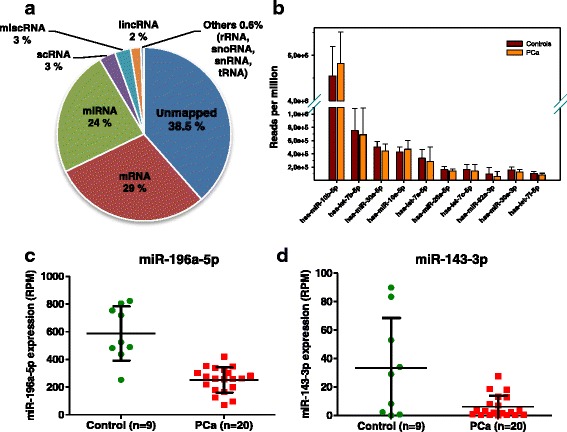
**a**. Pie chart showing the percentage of the reads mapped and unmapped to the genome. **b**. Top 10 highly expressed miRNAs in urinary exosomes. Amount (reads per million, RPM) of **c**. miR-196a-5p and **d**. miR-143-3p in 9 healthy controls and 20 PCa patients

In this study, RNA isolated from urinary exosomes from 20 PCa patients and 9 healthy male donors was used (Table 1). In order to optimize sequencing performance, the libraries were built using specific adaptors designed for small amounts of starting material (lower limit 1 ng total RNA). The libraries were PCR amplified, pooled and size selected and NGS was performed using Illumina high-throughput RNA sequencing technology. In average 5.8 million raw reads per sample were obtained and approximately 60% of the reads were mapped to the genome (Additional file 1: Table S1), mainly to miRNA (24%) and mRNA (29%) (Fig. 1a). The rest of the reads mapped to scRNA, miscRNA, and lincRNA, and rRNA, snoRNA, snRNA and tRNA together made 0.6% of the reads (Fig. 1a). The miRNA read counts normalized to reads per million (RPM) were used in further analyses.

**Table 1:** Clinical characteristics of patients in the NGS and in the PCR cohort. In the NGS cohort 10 patients had Gleason score 7a (3 + 4) and 10 patients had Gleason score 7b (4 + 3), and 13 and 7 patients were classified as intermediate and aggressive risk, respectively, using the D’Amico Risk Classification

		Discovery cohort (RNA-seq)		Validation cohort (RT-qPCR)	
		Control	Patient	Control	Patient
Number		9	20	19	28
Age (median, years)		58	66	56	67
Gleason Score^a^		nd		nd	
	7		20		21
	8–10				7
Clinical T-staging^b^		nd		nd	
	T1		11		12
	T2		6		9
	T3		3		7
PSA^b^ (ng/mL)		nd	8.8	nd	13.5

*Nd* non determined

^a^Gleason Score in biopsy tissue. ^b^Clinical T-staging and PSA levels (ng/mL) at time of diagnosis

As shown in Fig. 1b, the most abundant miRNAs was miR-10b-5p, followed by let-7b-5p, miR-30a-5p, miR-10a-5p and let-7a-5p. miR-10b-5p has also been shown to be very abundant in other studies of urinary exosomes [15, 16]. miRNAs with less than 10 reads per million in less than 5 samples were filtered out, leaving 254 miRNAs (Additional file 1: Table S2), or 217 miRNAs if miRNAs from different precursors were considered as one. In total 80 miRNA with 10 or more reads were found in all the samples. Hierarchical clustering analysis showed that the samples did not cluster into specific groups (Additional file 1: Figure S2). However, the level of 5 miRNAs was significantly different in control *versus* PCa patients: miR-196a-5p, miR-34a-5p, miR-143-3p, miR-501-3p and miR-92a-1-5p (Table 2, Fig. 1c, d, Additional file 1: Figure S3). All of these miRNAs were shown to be down-regulated in urinary exosomes from PCa patients. The most promising miRNA, miR-196a, was able to distinguish the two groups with 89% specificity and 100% sensitivity. Receiver operating characteristic (ROC) curve analysis showed that the area under the curve (AUC) for this miRNA was 0.92 (95% CI 0.79–1.06) and for miR143-3p was 0.72 (95% CI 0.48–0.97) (Additional file 1: Figure S4), thus showing the diagnostic potential of these miRNAs for PCa. Finally, no relevant differences were observed when PCa patients were subdivided by GS (GS 7a and GS 7b) or as intermediate and aggressive based on the D’Amico classification (see as an example miR-196a in Additional file 1: Figure S5).

**Table 2:** miRNAs significantly changed between PCa patients and healthy controls. The table shows miRNAs that were significantly changed compared to healthy controls in one of more of the following groups: all patients together, PC patients with Gleason score 7a (3 + 4), PC patients with Gleason score 7b (4 + 3), PC intermediate (D’Amico criteria) and PC aggressive (D’Amico criteria). Fold change: patient versus controls. Both SAM and rank product were used as statistical analysis

miRNAs	Fold change	q-value	Statistics
miR-196a-5p	−2.375	<0.05	SAM
miR-34a-5p	−4.385	<0.05	SAM
miR-501-3p	−7.315	0.009	Rank Product
miR-92a-1-5p	−6.481	0.025	Rank Product
miR-143-3p	−5.086	0.028	Rank Product

All of the 5 miRNAs shown to be significantly altered in PCa samples versus healthy controls in this study were downregulated in urinary exosomes of PCa patients. It is not clear whether the downregulation of these miRNAs is due to miRNA downregulation in the tumor or to nonspecific responses to the presence of the tumor. It has been suggested that, at least at early tumor stages, a reduction in the level of a circulating miRNA is probably not the result of a down regulation of the miRNA in the tumor itself, but to a negative effect of the tumor in the expression of the miRNA in other cells [17]. In a previous NGS of urinary exosomes, the majority of the miRNAs significantly altered between the control and the PCa group were also found to be downregulated, but the entire list of altered miRNA was not provided [16]. It is not clear why more miRNAs were altered between the control and the PCa group in that study compared to our study, but the different patient cohorts used or different NGS pipelines could be possible explanations. Interestingly, in the above mentioned study, PCR validation studies of 3 specific miRNAs (miR-21, miR-204 and miR-375) showed that only isomiRs (miRNA isoforms) with 3′ end modifications, but not the mature miRNAs, were able to discriminate between controls and PCa patients [16]. Since we only used the mature forms in our experimental PCR settings, it would be interesting to analyze potential differences in miRNAs isoforms in future studies. Finally, it should be mentioned that urine contains several population of exosomes, and that it is not clear to which extent prostate cells contribute to the total population. It is possible that if prostate-derived exosomes are isolated and analyzed separately, both upregulated and downregulated miRNAs are found in PCa patients.

### Validation of the RNA-seq data by quantitative PCR analysis

**Fig. 2:**
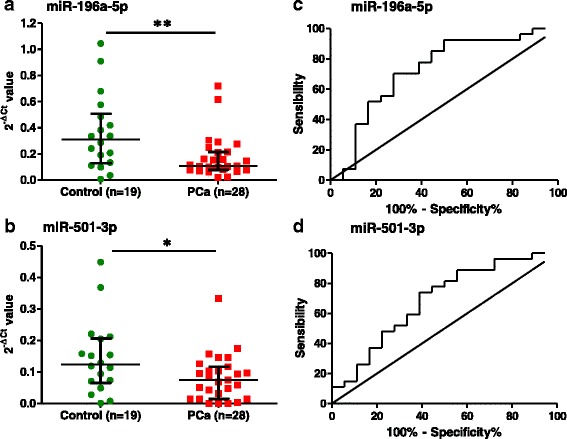
RT-qPCR analysis of selected miRNAs. **a**. miR-196a-5p and **b**. miR-501-3p were analyzed in 19 healthy controls and 28 PCa patients. The data was normalized to the average of three reference genes (miR-10b-5p, let-7b-5p and U6 snRNA). *P < 0.05; **P < 0.01 versus control group. **c**, **d**. ROC curves for miR-196a-5p (AUC = 0.73, 95% CI 0.56 to 0.89) and miR-501-3p (AUC = 0.69, 95% CI 0.52 to 0.85). AUC, area under the curve; CI, confidence interval

To validate the miRNAs identified by NGS, they were analyzed by quantitative RT-PCR on a new patient set including urine samples from 28 PCa patients and 19 healthy male donors (Table 1). For normalization we used the average of three miRNAs/small RNAs; miR-10b-5p, let-7b-5p and U6 snRNA. miR-10b-5p and let-7b-5p were chosen because, as shown in the NGS study, they were similarly expressed in the control and in the patient group (Additional file 1: Figure S6), and U6 snRNA was chosen as it has previously been used for normalization in other studies [18]. Importantly, the level of miR-196a-5p and miR-501-3p was significantly reduced in the PCa group compared to the healthy control group, thus validating the sequencing data by an additional analytical method in an independent sample set (Fig. 2a, b). ROC curves for these miRNAs showed that the AUC for miR-196-5p and miR-501-3p was 0.73 (95% CI 0.56–0.89) and 0.69 (95% CI 0.52–0.85) respectively (Fig. 2c, d). For comparison, it has been reported that the AUC for PSA to discriminate between any PCa and cancer-free controls in the Prostate Cancer Prevention Trial was 0.68 (95% CI 0.67–0.69) [19]. A combination (sum) of the two miRNAs did not significantly improve the diagnostic potential (AUC 0.74; 95% CI 0.59 to 0.89). Finally, we could not find a significant reduction of miR-34a-5p and miR-92a-1-5p level in the PCa group by RT-qPCR (Additional file 1: Figure S7), and miR-143-3p was not detected in the experimental conditions that were used.

Based on both NGS and PCR studies, miR-196a-5p appears as the most promising biomarker. The diagnostic performance calculated from NGS results (0.92; 95% CI 0.79–1.06) was better than from PCR results (0.73; 95% CI 0.56–0.89). This is often observed when biomarkers are measured by different methodologies, but the fact that both methods show a downregulation of miR-196a-5p opens for additional studies and optimizations. miR-196a has been found to be dysregulated, mainly upregulated, in several cancer types, and to act as an oncogene by affecting cell proliferation, migration and invasion [20, 21]. In fact, not much is known about miR-196a in PCa, though it has been shown that miR-196a is upregulated in PCa tissue compared to normal tissue [22, 23], while miR-196b has been shown to be downregulated [24]. However, it should be mentioned that exosomal miRNAs do not necessarily represent the cellular levels since miRNA sorting mechanism may affect the incorporation of miRNA into exosomes.

Among the numerous targets of miR-196a we find Radixin, ANXA1, several HOX genes and ETS-related gene (ERG). Remarkably, ERG is the most frequently overexpressed oncogene in PCa, and high expression of ERG is associated with advanced tumour stage, high Gleason score, metastasis and shorter survival times [25]. The oncogene function of ERG is related to the regulation of a plethora of genes implicated in several cellular processes such as differentiation, growth, motility and invasion [25]. It is possible then that the high levels of miR-196a-5p in normal prostate cells help to maintain the levels of ERG low. In terms of PCa, it is also interesting to find that analysis of miR-196a-5p in DIANA-miRPath v3.0 identified 16 target genes belonging to the KEGG pathway *Prostate cancer (hsa05215)* (Additional file 1: Figure S8) [26]. However, when looking for potential targets of the downregulated miRNAs that may be relevant for PCa we should keep in mind that, as previously mentioned, the expression of the miRNAs is not necessarily changed in the tumor.

miR-501-3p was also shown to be downregulated in urinary exosomes from PCa patients both by NGS and by PCR. It has recently been shown that miR-501-3p promoted the invasiveness of pancreatic ductal adenocarcinoma cells possibly by suppressing E-cadherin [27]. Downregulation of miR-501-3p in serum has recently been found to be useful for prediction and prognosis of lymph node metastasis in gastric cancer [28], but miR-501-3p has been found to be upregulated in serum samples of PCa patients compared to benign prostatic hyperplasia [29].

## Conclusion

The discovery of biomarkers that can supplement PSA is a main goal of PCa research. In particular, biomarkers for early diagnosis and risk stratification are urgently needed. This study confirms the feasibility of NGS analysis of small amount of exosomal RNA and shows that NGS is a valid method to identify novel RNA-based biomarkers for PCa. We have shown that miR-196a-5p and miR-501-3p have diagnostic potential for PCa. These miRNAs will have to be the subject of future studies in order to determine their specific clinical utility in additional patient cohorts. Furthermore, the functional role of these miRNA in relation to PCa is also an interesting subject that requires further study.

## Additional file

Additional file 1: Materials and methods; Table S1 and S2; Supplementary figures S1, S2, S3, S4, S5, S6, S7, S8. (PDF 457 kb)

## Abbreviations

AUC: Area under the curve
ERG: ETS-related gene
GS: Gleason score
miRNA: microRNA
NGS: Next Generation Sequencing
PCa: Prostate cancer
PSA: Prostate specific antigen
ROC: Receiver operating curve
