# Supplementary material for: Identification of non-invasive miRNAs biomarkers for prostate cancer by deep sequencing analysis of urinary exosomes
Source: Mol Cancer. 2017 Oct 5;16:156. doi: 10.1186/s12943-017-0726-4 (PMC5629793; doi:10.1186/s12943-017-0726-4)
Supplement: Additional file 1: — Materials and methods; Table S1 and S2; Supplementary figures S1, S2, S3, S4, S5, S6, S7, S8. (PDF 457 kb) [file 12943_2017_726_MOESM1_ESM.pdf]

Supplementary information

- Materials and methods

- Supplementary Table legends

Table S1. Number of total reads, and reads mapped and unmapped to the genome per sample.

Table S2. Total list of miRNAs identified by miRDeep2 per sample. MiRNAs with less than 10 reads per million in less than 5 samples are not included. C: control; P: Prostate cancer patient.

- Supplementary Figure legends

Figure S1. Typical profile of RNA from urinary exosomes in Agilent RNA 6000 Pico chips.

Figure S2. Hierarchical clustering analysis of the miRNAs identified in urinary exosomes. Blue: healthy controls. Pink: Prostate cancer patients with Gleason score 7a (3+4). Green: Prostate cancer patients with Gleason score 7b (4+3).

Figure S3. Amount (reads per million, RPM) of A. miR-501-3p, B. miR-92-1-5p and C. miR-34a-5p in 9 healthy controls and 20 PCa patients.

Figure S4. ROC curves for A. miR-196a-5p (AUC=0.92, 95% CI 0.7905 to 1.065) and B. miR-143-3p (AUC=0.72, 95% CI 0.4784 to 0.9660). AUC, area under the curve; CI, confidence interval.

Figure S5. Boxplots showing RNA-sequencing expression (reads per million, RPM) of miR196a-5p. A. Differences between healthy controls, PCa Gleason score 7a and PCa Gleason score 7b. B. Differences between healthy control, PCa intermediate and PCa aggressive as evaluated following D'Amico criteria.

Figure S6. Boxplots showing the RNA-sequencing expression (reads per million, RPM) of the microRNAs used for normalization of the qPCR results. A. miR-10b-5p. B. Let-7b-5p.

Figure S7. RT-qPCR analysis of A. miR-34a-5p and B. miR-92a-1-5p in 19 healthy controls and 28 PCa patients. Data was normalized to the average of three reference genes (miR-10b-5p, let-7b-5p and U6 snRNA).

Figure S8. Analysis of miR-196a-5p in DIANA-miRPath v3.0 [26] identified the KEGG pathway *Prostate cancer (hsa05215)* with a p-value under 0.05. The 16 genes of the pathway are indicated in yellow boxes: BRAF, E2F2, NRAS, CDK2, HSP90AA1, RAF1, CDKN1B, IGF1R, CCND1, CCNE2, HSP90AB1, NFKBIA, AKT3, FOXO1, MAPK1, MDM2.

MATERIALS AND METHODS

Urine Samples

Urine samples (29 samples for NGS analysis and 47 samples for RT-qPCR validation) were collected from healthy controls and PCa patients approximately 1 week before prostatectomy as previously described [1,2]. The pH in urine samples and the presence of leucocytes, nitrites, proteins, glucose, ketones and blood were analysed with a Combur-Test strip in an Urysys 1100 urine analyser (Roche Diagnostics, Basel, Switzerland). The collection of urine samples was approved by the Norwegian Regional Committees for Medical and Health Research Ethics and the participants gave informed written consent.

Isolation of urinary exosomes

Exosomes were isolated from fresh urine samples by differential centrifugation as previously described [2]. Briefly, urine samples (in general 50-150 mL) were centrifuged at 2,000 *g* for 15 min at room temperature (RT), and then at 10,000 *g* for 30 min at RT to remove cell debris and large particles. The supernatant was then centrifuged at 100,000 *g* for 70 min at RT in a Ti70 rotor (fixed angle), and the pellet was washed with phosphate-buffered saline (PBS) and pelleted again at 100,000 *g* for 70 min at 4 °C in a Ti70 rotor. Exosomes were then resuspended in PBS, vortexed, filtrated through a 200-nm pore Supor syringe filter (Pall, Port Washington, NY, USA) and finally pelleted at 100,000 *g* for 70 min at 4 °C in a SW40 rotor (swinging bucket). The supernatant was removed leaving 50–100 μL in the bottom to resuspend the pellet. Exosomes were submitted to several analyses to control the purity and yield, and they were then stored at –80 °C for subsequent applications.

Protein measurements

The amount of protein in exosomes was determined using a bicinchoninic acid (BCA) assay kit (Thermo Scientific, Rockford, IL, USA) according to the manufacturer´s instructions. Bovine serum albumin was used as standard protein.

RNA extraction of urinary EVs

Samples of urinary exosomes (approximately 5 ug protein as measured with the BCA assay) were treated with Proteinase K (Sigma-Aldrich) for 60 min and RNase A (Roche Diagnostics) for 15 min. Total RNA was extracted from exosomes using a miRNeasy Micro Kit (Qiagen) according to the manufacturer´s instructions and eluted in 15 µL of RNAse-free water. After that, total RNA was treated with DNase (Ambion) for 15 min. The extracted RNA was run in an Agilent 2100 Bioanalyzer using a RNA 6000 Pico Kit (Agilent Technologies) to detect potential degradation and ribosomal contamination.

Next generation sequencing

NGS was performed at the Genomics Core Facility Oslo (The Norwegian Radium Hospital, Norway). Small RNA libraries were prepared using the CleanTag Small RNA library prep kit (TriLink Biotechnologies, USA). Total RNA (1-4 ng) was ligated to 3'- and 5'- RNA adapters, and reversely transcribed to generate cDNA libraries for each sample. Libraries were PCR amplified, pooled and size selected using acrylamide gel (6%, Novex TBE Gel, Thermo Fisher Scientific) purification before and then quantified by real-time PCR. Small RNA libraries were sequenced single read 50 base pairs using a NextSeq500 (Illumina) instrument. Real-time analysis, base calling and filtering of low quality reads were done by Illumina's software packages (SCS2.9/RTA1.9 and Off-line Basecaller v1.9). Fastq files for each sample were analysed using the software package miRDeep2 to map the sequencing reads to the human genome (hg19), identify miRNAs (miRBase, http://www.mirbase.org/) and normalize the expression values (read count normalization) for expression profiling across samples.

RT-qPCR

cDNA synthesis from exosomal RNA was performed using miRCURY LNA Universal RT microRNA PCR, polyadenylation and cDNA synthesis kit (Exiqon) according to the manufacturer´s protocol. A C1000 thermal cycler (Bio-Rad, Oslo, Norway) running for 60 min at 42 °C and for 5 min at 95 °C was used. A RNA spike-in provided with the kit was added to the RNA samples as a control. Real-time qPCR was performed using a LightCycler 480 (Roche Applied Science, Mannheim, Germany) at the following conditions: 95 °C for 10 min, thereafter 45 amplifications cycles at 95 °C for 10 s, and 60 °C for 1 min (ramp-rate 1.6 °C/s). The target sequences (5´-3´) of the primer sets were:
miR-196a-5p (UAGGUAGUUUCAUGUUGUUGGG),
miR-34a-5p (UGGCAGUGUCUUAGCUGGUUGU),
miR-143-3p (UGAGAUGAAGCACUGUAGCUC),
miR-501-3p (AAUGCACCCGGGCAAGGAUUCU),
miR-92a-1-5p (AGGUUGGGAUCGGUUGCAAUGCU),
miR-10b-5p (UACCCUGUAGAACCGAAUUUGUG),
let-7b-5p (UGAGGUAGUAGGUUGUGUGGU),
U6 snRNA(GUGCUCGCUUCGGCAGCACAUAUACUAAAAUUGGAACGAUACAGAG AAGAUUAGCAUGGCCCCUGCGCAAGGAUGACACGCAAAUUCGUGAAGCGUUCCAUA UUUUU).
miRNAs with C_q values above 37 were considered as not expressed.

Statistical analysis

The NGS data were analyzed in J-Express 2011. In the analysis, only miRNAs with more than 10 reads in at least 5 samples were included. The filtered dataset was then Log2-transformed. Dataset used for unsupervised two-way hierarchical clustering was in addition mean normalized prior to clustering. Two statistical analysis were performed, the rank product (20,000 permutations) [3] and SAM (Significance analysis of microarrays) (20,000 permutations) [4]. For analysis of RT-qPCR data the comparative Cq method was used, using miR-10b-5p, miR-let7b-5p and U6 snRNA for normalization. Statistical analysis was executed using a Mann–Whitney *U* test. A P-value<0.05 was considered as significant. Receiver operating characteristic (ROC) curves were done in Prism V (Graphpad Software).

References

1. Skotland T, Ekroos K, Kauhanen D, Simolin H, Seierstad T, Berge V, et al. Molecular lipid species in urinary exosomes as potential prostate cancer biomarkers. Eur J Cancer. 2017;70:122–32.

2. Øverbye A, Skotland T, Koehler CJ, Thiede B, Seierstad T, Berge V, et al. Identification of prostate cancer biomarkers in urinary exosomes. Oncotarget. 2015;6:30357–76.

3. Breitling R, Armengaud P, Amtmann A, Herzyk P. Rank products: a simple, yet powerful, new method to detect differentially regulated genes in replicated microarray experiments. FEBS Lett. 2004;573:83–92.

4. Tusher VG, Tibshirani R, Chu G. Significance analysis of microarrays applied to the ionizing radiation response. Proc Natl Acad Sci. 2001;98:5116–21.

Sample Type	Total	Mapped	Unmapped	% mapped	% unmapped
C1	14744662	4939713	9804949	33.5%	66.5%
C2	25069160	5694612	19374548	22.7%	77.3%
C3	6097126	2949820	3147306	48.4%	51.6%
C4	5077743	2577090	2500653	50.8%	49.2%
C5	5740963	4345992	1394971	75.7%	24.3%
C6	6529973	3449163	3080810	52.8%	47.2%
C7	3699322	2545515	1153807	68.8%	31.2%
C8	3324769	2179708	1145061	65.6%	34.4%
C9	4595931	3216591	1379340	70 %	30 %
P1	5042205	3186596	1855609	63.2%	36.8%
P2	4775506	2627659	2147847	55 %	45 %
P3	5132138	3372857	1759281	65.7%	34.3%
P4	5179459	2905727	2273732	56.1%	43.9%
P5	2955057	2224494	730563	75.3%	24.7%
P6	4181980	2511715	1670265	60.1%	39.9%
P7	4149457	3209495	939962	77.3%	22.7%
P8	1983881	1449041	534840	73 %	27 %
P9	4222884	3012547	1210337	71.3%	28.7%
P10	3088721	2281632	807089	73.9%	26.1%
P11	6878342	3940850	2937492	57.3%	42.7%
P12	42596844	7589966	35006878	17.8%	82.2%
P13	8541862	3999053	4542809	46.8%	53.2%
P14	5315745	3782073	1533672	71.1%	28.9%
P15	4894333	3383056	1511277	69.1%	30.9%
P16	4655944	2762665	1893279	59.3%	40.7%
P17	5212260	4059364	1152896	77.9%	22.1%
P18	4752999	3896576	856423	82 %	18 %
P19	2544154	1775456	768698	69.8%	30.2%
P20	3956526	2883126	1073400	72.9%	27.1%

- Table S1 -

#miRNA	C1	C2	C3	C10	C5	C6	C7	C8	C9	P1	P2	P3	P4	P5	P6	P7	P8	P9	P10	P11	P12	P13	P14	P15	P16	P17	P18	P19	P20	precursor
hsa-let-7a-5p	43445.1	61968.5	30341.9	33693.9	21264.6	33524.5	22415.5	33367.6	18207.3	18876.0	23506.8	12499.6	34728.8	20408.9	29456.4	15602.6	16848.2	25149.8	22725.8	23261.6	116629.9	40964.1	22103.2	20565.0	31620.9	22067.4	20213.5	27098.7	24590.4	hsa-let-7a-1
hsa-let-7a-5p	43525.4	62056.7	30400.8	33767.3	21303.2	33595.4	22512.0	33404.9	18223.9	18923.4	23564.2	12552.8	34799.1	20447.4	29517.0	15625.0	16967.0	25195.6	22847.3	23297.4	116744.0	41033.5	22133.4	20619.2	31709.0	22099.9	20238.4	27216.8	24642.7	hsa-let-7a-2
hsa-let-7a-5p	44214.9	63094.2	31042.2	34961.8	22027.7	34515.1	23073.8	34704.5	18742.8	19289.9	24041.1	12838.8	35633.9	21246.0	30355.0	16255.8	17500.4	25952.7	23818.5	23907.3	118238.9	41861.8	22996.0	21366.6	33038.8	22911.8	20702.7	27657.1	25280.9	hsa-let-7a-3
hsa-let-7b-5p	104060.1	147858.8	73869.9	47914.8	45375.9	78744.0	61529.4	67738.4	49346.1	52459.1	53332.1	33799.6	88101.4	55841.9	80501.0	45317.9	37252.4	56344.3	51425.1	74890.1	226376.2	81296.7	60854.4	65356.5	89436.8	66537.7	50876.4	58368.5	50264.9	hsa-let-7b
hsa-let-7c-5p	23893.7	32887.8	12243.5	9790.0	9597.1	17335.7	12424.8	19888.1	8955.1	12419.4	12909.6	7275.7	14143.8	12173.5	14941.2	9297.1	6620.0	15572.4	11681.6	15316.3	53049.7	18628.5	12907.1	12513.8	10936.3	15308.6	7661.8	11049.7	9966.5	hsa-let-7c
hsa-let-7d-3p	247.3	317.3	238.4	286.0	192.9	415.5	303.2	259.6	197.4	213.0	198.2	43.6	161.9	222.3	249.4	149.7	211.2	212.5	152.9	219.2	112.2	147.0	256.9	260.7	205.6	214.5	201.5	115.5	127.2	hsa-let-7d
hsa-let-7d-5p	1815.5	2363.5	1274.5	1186.2	865.4	1683.3	819.3	1279.0	888.3	690.3	849.9	408.1	1173.2	792.7	1149.6	608.1	633.5	1025.1	695.5	851.4	3060.9	1514.6	876.1	856.6	1229.6	860.3	825.4	855.9	876.7	hsa-let-7d
hsa-let-7e-5p	3963.7	5281.0	3630.9	1877.2	2101.9	4563.9	3505.5	3869.1	2711.3	2059.2	1970.2	1462.4	3109.4	2496.7	3793.4	1679.6	1772.5	2729.6	2104.0	2931.9	3326.0	3270.9	2904.6	2668.3	3114.3	3275.7	2614.1	2609.7	2527.8	hsa-let-7e
hsa-let-7f-5p	13067.8	15764.4	9498.2	10322.0	8570.2	12909.7	8836.5	9914.3	6440.9	6402.7	7503.1	5449.1	8997.2	8243.8	9624.8	5637.9	5388.6	7709.9	6599.5	8063.2	15719.7	12154.4	9267.4	7760.5	10988.3	8241.1	8748.1	7653.4	7370.1	hsa-let-7f-1
hsa-let-7f-5p	13130.0	15781.0	9485.0	10252.3	8594.9	12927.1	8831.6	9904.2	6433.9	6364.8	7486.9	5442.3	8987.8	8258.2	9625.6	5646.7	5280.7	7682.7	6575.6	8073.3	15735.4	12069.2	9301.5	7755.9	10974.1	8246.2	8745.8	7709.8	7381.7	hsa-let-7f-2
hsa-let-7g-5p	805.6	1557.8	565.9	924.6	439.7	653.9	963.4	1163.7	385.8	475.5	529.6	138.5	569.9	412.0	492.6	334.1	208.6	574.7	338.5	435.9	2321.0	1012.4	592.0	453.0	528.8	449.5	523.7	489.2	532.1	hsa-let-7g
hsa-let-7i-5p	3495.6	3719.9	3085.6	2339.1	2517.7	2830.2	2926.6	4479.9	2389.8	2719.8	2751.5	2475.1	2665.6	2506.0	3767.5	2544.4	2237.4	2897.8	1715.5	2463.2	4040.7	2925.0	2373.5	3921.5	2598.7	3355.7	2711.7	2664.4	1966.1	hsa-let-7i
hsa-miR-100-5p	175.0	158.7	134.0	227.2	95.6	122.8	82.4	170.1	74.9	37.6	164.1	39.4	58.1	52.9	60.2	44.2	142.7	83.3	87.6	138.1	82.4	176.2	63.0	26.8	53.4	39.2	45.9	47.8	43.0	hsa-mir-100
hsa-miR-101-3p	711.0	578.2	828.8	805.9	356.1	334.7	408.5	582.7	285.8	766.7	796.1	889.7	514.9	372.2	325.0	556.5	445.6	441.0	477.7	928.7	359.7	660.9	465.8	504.9	581.5	365.4	362.5	468.8	423.2	hsa-mir-101-1
hsa-miR-101-3p	730.1	598.7	850.0	855.7	381.9	334.7	432.9	647.2	291.9	781.5	825.9	915.3	528.5	415.8	339.9	627.8	445.6	477.9	517.4	949.2	366.4	687.8	521.3	504.9	655.0	394.4	404.7	469.3	441.8	hsa-mir-101-2
hsa-miR-103a-3p	1869.3	3163.6	1375.7	1384.6	1064.3	2850.1	2077.9	2190.6	1407.4	989.5	1184.2	790.3	1685.6	1467.7	2098.9	1342.3	1625.3	1924.0	1305.9	1310.2	4320.4	1511.0	1874.6	1711.3	3557.5	1829.5	1921.0	1269.9	1572.6	hsa-mir-103a-1
hsa-miR-103a-3p	1869.5	3163.6	1375.7	1394.7	1074.2	2850.1	2077.9	2190.6	1407.4	989.5	1184.2	790.3	1685.6	1483.8	2098.9	1342.3	1625.3	1924.0	1306.4	1310.2	4336.9	1522.5	1874.6	1711.3	3568.2	1829.5	1921.0	1289.8	1580.6	hsa-mir-103a-2
hsa-miR-106b-3p	37.7	136.3	54.3	22.5	54.1	104.8	70.2	79.4	94.8	61.0	43.1	70.6	47.0	58.9	70.0	56.3	197.6	72.7	48.4	27.8	224.8	50.8	63.2	112.1	45.8	90.3	86.4	53.2	72.9	hsa-mir-106b
hsa-miR-107	307.7	611.9	298.5	242.2	162.4	598.4	314.3	378.4	118.5	215.4	181.0	170.0	292.8	165.6	301.0	104.6	249.3	257.0	206.6	195.4	762.6	261.9	244.0	192.3	320.4	221.0	181.5	233.0	296.0	hsa-mir-107
hsa-miR-10a-5p	47752.7	30423.9	43826.1	41958.6	43275.7	53358.6	42483.3	33140.9	49844.1	64169.9	53189.2	51480.9	32755.3	59835.9	44250.1	42067.9	41171.9	64466.8	39441.5	61241.0	3270.7	47241.4	45489.8	51006.5	41533.9	57347.1	50734.7	41921.1	46384.9	hsa-mir-10a
hsa-miR-10b-5p	375699.0	249858.9	496967.7	577786.3	641228.8	500173.2	588847.0	509664.1	646598.3	596126.7	583569.2	707731.8	512908.8	599605.2	528366.0	661874.1	677171.1	595565.9	669216.1	545863.5	41233.1	479133.2	611245.0	568380.2	490038.8	580986.7	585419.8	619746.4	634811.4	hsa-mir-10b
hsa-miR-125a-5p	439.4	440.9	396.2	194.7	134.2	198.2	151.8	78.9	169.3	397.6	371.6	303.1	221.8	226.6	235.3	164.1	144.6	185.7	159.5	348.5	127.1	312.4	168.8	212.8	149.8	135.0	286.5	68.7	127.5	hsa-mir-125a
hsa-miR-125b-2-3p	378.2	380.4	335.2	48.3	99.5	228.1	135.0	99.5	72.8	258.0	236.6	67.0	142.4	173.2	116.9	129.3	36.8	69.4	95.1	223.9	353.8	337.8	83.4	219.1	11.1	157.4	88.8	183.1	120.5	hsa-mir-125b-2
hsa-miR-1307-3p	276.0	624.1	326.2	255.3	211.8	459.7	281.5	249.5	231.4	235.8	240.9	90.1	308.1	72.0	194.8	85.7	186.0	109.5	85.6	393.3	285.2	290.6	249.5	296.4	340.5	172.1	174.3	92.9	124.5	hsa-mir-1307
hsa-miR-141-3p	159.0	320.3	142.7	71.1	153.8	79.2	239.1	280.6	137.5	149.0	208.6	146.6	215.5	130.0	152.3	197.7	153.0	259.3	94.6	223.9	257.3	294.7	63.5	168.6	506.6	174.4	384.4	117.6	217.1	hsa-mir-141
hsa-miR-146a-5p	61.2	162.9	108.6	17.6	67.2	82.9	73.2	132.4	25.5	28.4	33.0	48.1	115.9	26.3	221.5	206.5	78.8	75.2	46.3	40.3	129.1	176.2	81.4	30.0	2359.4	102.8	6545.0	322.7	147.8	hsa-mir-146a
hsa-miR-146b-5p	3154.2	2890.8	2731.8	2357.9	2527.0	2897.1	1820.5	2167.4	2321.1	2497.3	1768.4	1879.5	2477.6	2358.2	2200.5	2405.9	2081.8	2018.0	2042.4	2700.0	1293.3	2707.7	2249.1	1962.0	2176.0	1796.6	3811.5	1572.2	2471.9	hsa-mir-146b
hsa-miR-148a-5p	685.3	846.0	239.7	323.4	313.8	459.1	349.4	888.3	171.4	286.2	512.8	358.3	884.2	313.8	662.1	384.2	351.9	459.5	273.6	458.8	1184.6	621.4	232.8	554.0	593.0	308.3	181.2	519.8	553.1	hsa-mir-148a
hsa-miR-148a-3p	6773.7	9253.9	2980.2	2237.0	1649.4	2286.2	2288.5	3032.9	1268.5	3367.7	3984.6	2444.7	5834.8	2240.5	3524.7	2517.3	1383.8	1837.8	1863.0	3736.1	15662.8	5145.3	1972.5	3212.6	3530.4	1731.2	1182.3	1758.0	2699.0	hsa-mir-148a
hsa-miR-151a-3p	4205.0	4274.3	4187.2	2294.6	3323.0	5087.9	5075.8	3789.7	4475.2	3262.2	3671.5	2832.5	4211.6	4346.8	4670.4	4083.5	5102.5	5168.5	3602.1	3238.9	2421.9	3800.2	4108.3	4622.4	6428.4	4498.9	8018.5	3934.9	3980.2	hsa-mir-151a
hsa-miR-155-5p	413.5	1258.6	66.2	79.0	119.0	68.4	116.7	187.7	66.1	44.1	172.7	25.3	305.7	34.3	285.2	202.7	94.3	56.2	107.5	137.6	4582.9	138.4	29.9	174.9	690.8	77.0	224.1	317.4	296.0	hsa-mir-155
hsa-miR-181a-5p	11000.4	9702.2	15243.3	7930.0	7362.3	10382.9	10339.6	10286.1	6925.0	7027.5	13011.6	5636.0	6767.1	7624.3	9474.6	6133.7	8577.1	5411.7	7000.0	7323.5	1244.7	8026.3	6302.3	5914.6	9104.7	7044.9	9475.9	6372.2	4689.4	hsa-mir-181a-1
hsa-miR-181a-2-3p	96.7	345.5	157.1	168.5	113.8	162.3	242.2	98.7	48.8	95.7	200.7	121.0	199.8	98.7	198.3	127.0	71.0	85.9	195.9	289.8	112.2	188.6	151.1	356.0	269.4	129.5	91.6	182.6	45.3	hsa-mir-181a-2
hsa-miR-181a-5p	11000.4	9702.2	15243.3	7930.0	7362.3	10382.9	10339.6	10286.1	6925.0	7027.5	13011.6	5636.0	6767.1	7624.3	9474.6	6133.7	8577.1	5411.7	7000.0	7323.5	1244.7	8026.3	6302.3	5914.6	9104.7	7044.9	9475.9	6372.2	4689.4	hsa-mir-181a-2
hsa-miR-181b-5p	941.9	1358.4	1280.6	683.1	561.5	1070.0	810.9	1586.4	431.4	712.8	1149.0	568.0	1122.3	586.9	1052.0	482.6	821.4	516.0	625.7	804.3	402.9	813.4	560.4	761.4	648.8	700.4	894.1	584.8	355.3	hsa-mir-181b-1
hsa-miR-181b-5p	955.1	1377.5	1280.6	683.1	561.5	1070.0	820.8	1586.4	431.4	712.8	1149.0	568.0	1122.3	600.0	1052.0	482.6	821.4	516.0	625.7	804.3	415.0	824.4	560.4	770.2	648.8	716.3	896.7	605.2	355.3	hsa-mir-181b-2
hsa-miR-181c-5p	211.4	86.4	177.4	53.9	324.8	103.7	95.4	241.2	279.9	259.5	267.9	68.4	241.3	176.6	415.1	270.2	209.9	204.7	106.2	139.0	60.8	129.3	131.0	194.0	345.4	351.8	610.8	135.3	199.4	hsa-mir-181c
hsa-miR-181d-5p	107.4	117.6	67.8	116.0	142.4	26.8	103.7	141.2	114.1	132.4	129.3	89.8	70.0	122.4	188.5	140.5	91.7	135.9	132.7	132.0	45.1	80.2	50.5	46.5	137.3	140.8	184.0	96.1	31.0	hsa-mir-181d
hsa-miR-182-5p	4281.3	5683.8	3110.0	4836.6	2879.7	4052.3	4799.3	4957.8	3889.7	5348.7	3121.0	5858.9	5074.2	4420.9	4709.7	3717.8	2825.7	4064.3	4522.0	5403.0	8652.2	3000.4	3371.4	5181.5	5668.9	3578.5	5365.1	5341.8	3111.5	hsa-mir-182
hsa-miR-183-5p	1176.5	908.1	1558.9	2711.6	679.4	977.2	1234.2	952.8	537.3	2780.2	1511.3	2613.2	764.8	1049.8	1078.8	805.2	543.7	989.0	461.2	1930.9	1197.6	1370.5	896.2	909.4	1036.1	488.9	1321.6	1194.8	735.2	hsa-mir-183
hsa-miR-186-5p	365.1	533.1	214.3	181.6	205.6	300.5	206.0	361.3	108.2	198.8	137.5	82.8	205.7	144.8	237.2	188.0	221.5	306.4	109.9	194.4	491.5	270.3	112.3	273.6	176.2	214.5	267.7	110.1	107.2	hsa-mir-186
hsa-miR-191-5p	3161.6	3091.2	2381.9	1218.1	1312.8	2396.7	2912.5	1930.2	2174.9	1381.4	1442.0	929.6	2450.4	1812.4	2807.1	1794.3	1438.0	2253.0	1729.5	1888.4	2248.5	2696.4	1707.6	2201.9	3162.2	1865.9	4396.2	1494.4	1877.9	hsa-mir-191
hsa-miR-192-5p	2203.5	2416.6	1868.7	785.7	875.0	1372.5	1280.8	1639.0	461.8	691.4	1466.8	483.0	829.6	804.1	869.5	871.5	738.1	641.6	908.0	657.0	653.9	1438.2	1209.4	774.5	1125.6	553.2	579.6	620.2	510.8	hsa-mir-192
hsa-miR-196a-5p	716.0	609.9	726.3	232.1	421.2	630.0	466.9	374.5	438.4	208.6	126.4	119.9	304.6	268.9	260.5	206.5	51.0	234.4	275.2	289.1	209.9	292.8	390.1	223.1	314.9	242.4	158.0	85.9	145.8	hsa-mir-196a-1
hsa-miR-196a-5p	930.2	897.5	886.0	274.4	557.8	808.9	581.7	502.1	525.6	228.7	205.4	130.6	392.7	337.5	344.6	318.2	89.1	287.0	289.7	359.8	304.8	382.8	450.4	292.7	390.8	309.7	199.4	105.8	251.1	hsa-mir-196a-2
hsa-miR-200a-5p	65.4	155.3	113.1	74.9	89.1	219.9	111.8	100.9	59.4	86.5	45.2	29.8	133.0	84.3	230.9	60.4	153.7	45.2	55.8	104.2	224.4	86.4	89.9	141.8	143.6	39.4	155.4	90.2	34.3	hsa-mir-200a
hsa-miR-200a-3p	896.5	1171.2	849.3	466.4	669.9	1287.9	1096.6	858.5	841.5	801.9	699.8	447.5	787.5	775.8	1021.0	708.6	642.5	1310.2	320.3	792.2	636.7	953.0	711.0	1083.1	1655.5	1101.3	1534.5	689.5	912.3	hsa-mir-200a
hsa-miR-200b-3p	249.9	457.2	108.9	18.3	88.9	239.5	164.0	128.9	162.3	150.2	77.2	67.8	332.8	155.0	295.1	150.0	60.7	206.3	133.9	96.6	933.2	120.7	125.2	221.7	120.7	216.1	255.8	126.7	157.5	hsa-mir-200b
hsa-miR-200c-3p	2874.5	4333.2	1541.2	1166.8	1192.9	3960.9	3510.9	2263.4	2159.9	1534.8	1228.7	1068.9	3011.6	2541.2	3500.7	1789.0	1996.6	3038.2	2222.2	1592.2	4666.4	2460.6	2428.6	3525.0	7249.5	2524.4	4158.3	2212.3	2965.0	hsa-mir-200c
hsa-miR-203a-3p	587.8	780.8	541.8	259.8	250.9	857.3	1204.9	453.8	375.8	439.9	441.3	146.9	985.2	431.1	855.3	605.7	1142.9	1135.2	359.1	423.7	518.2	645.1	726.5	726.8	1214.0	508.2	838.7	861.8	918.6	hsa-mir-203a
hsa-miR-204-5p	3430.8	5399.4	2872.6	2444.0	3762.2	3440.0	3259.5	4541.2	2711.3	2242.6	2308.5	1405.8	5594.3	2436.1	4961.9	2505.5	2907.0	2129.0	1762.6	3435.7	4592.3	4467.6	2962.3	4236.1	3242.6	4402.8	2267.4	4619.0	2453.9	hsa-mir-204
hsa-miR-205-5p	81.5	296.6	157.8	69.6	12.0	167.5	175.8	101.7	32.5	84.1	93.4	52.9	151.8	72.0	164.9	42.7	145.9	170.2	76.0	26.9	243.6	105.6	35.4	79.9	291.6	75.6	415.6	110.1	51.6	hsa-mir-205
hsa-miR-21-5p	3941.3	8137.1	1968.9	1918.8	2101.3	5312.1	5891.6	11676.0	2060.5	1749.3	2088.3	1341.7	10793.8	3000.2	10473.5	4464.4	2928.4	5787.8	3146.7	1789.2	17018.5	4948.0	4887.5	6489.1	27876.5	3748.7	22518.9	6164.4	4787.3	hsa-mir-21
hsa-miR-22-3p	1649.7	1749.0	1718.6	680.5	597.1	1236.4	963.8	1012.0	733.5	835.1	652.8	507.2	967.1	732.2	899.4	798.4	488.2	677.4	334.3	948.2	1201.1	1208.2	614.4	876.9	1034.4	865.6	1019.7	526.8	551.4	hsa-mir-22
hsa-miR-221-3p	671.5	968.2	260.6	285.2	462.9	755.0	459.6	420.1	361.8	240.0	383.8	229.4	479.7	152.9	440.3	165.9	237.0	738.4	195.1	424.9	523.7	387.3	942.1	576.2	956.7	590.0	306.6	326.5	391.9	hsa-mir-221
hsa-miR-222-3p	668.2	740.3	326.2	564.1	340.9	471.7	379.1	260.0	496.0	453.3	403.6	201.2	343.9	129.2	389.1	195.0	304.8	346.4	233.9	665.0	298.9	340.4	711.0	464.4	535.8	368.6	224.6	293.7	276.7	hsa-mir-222
hsa-miR-25-3p	958.7	1693.9	520.6	698.9	439.9	1436.9	802.1	1424.2	395.4	395.5	527.5	255.8	1230.3	628.8	771.1	422.2	322.2	729.7	365.8	406.0	3487.6	679.6	625.6	825.3	1446.0	801.1	675.1	581.5	370.3	hsa-mir-25
hsa-miR-26a-5p	24388.7	21129.4	20605.0	9156.7	12673.1	15471.9	14675.9	12926.1	15032.4	14771.3	12577.8	9471.5	13840.9	15717.0	16231.7	13392.6	9641.9	15562.7	9180.8	14146.5	17654.7	16518.1	13539.7	17805.1	17494.0	16197.5	18148.3	12415.8	15259.9	hsa-mir-26a-1
hsa-miR-26a-5p	24388.7	21129.4	20605.0	9156.7	12673.3	15491.3	14675.9	12926.1	15032.4	14771.3	12578.2	9471.5	13840.9	15717.4	16231.7	13392.6	9641.9	15562.7	9180.8	14146.5	17655.1	16518.1	13539.7	17805.1	17494.0	16197.5	18148.3	12415.8	15259.9	hsa-mir-26a-2
hsa-miR-26b-5p	4040.2	6926.0	2349.7	1070.6	1639.9	2601.2	2195.8	2962.7	1814.8	1772.4	1479.0	772.6	2864.0	1488.0	2965.7	1672.0	1094.5	2200.1	1423.3	1994.2	10267.6	2571.7	1730.0	3030.6	2550.8	2632.7	2713.3	1678.0	1811.0	hsa-mir-26b
hsa-miR-27b-3p	299.3	286.6	232.3	140.0	186.7	225.0	256.7	155.2	187.5	168.9	120.7	26.7	217.2	208.3	150.7	176.8	134.3	213.1	142.2	141.9	139.3	132.1	88.9	192.0	134.6	189.8	161.0	149.8	88.2	hsa-mir-27b
hsa-miR-28-3p	4400.1	5798.5	3651.5	3108.0	2131.6	3072.9	3256.5	2598.8	2457.7	3304.9	3920.7	2963.9	3628.1	2900.7	3204.9	2603.0	3031.7	2493.2	2566.4	3558.7	4595.0	4092.2	2601.6	3216.9	4841.2	2395.6	4061.2	1802.6	2398.3	hsa-mir-28
hsa-miR-30a-3p	13399.2	16081.5	13298.8	9358.4	15411.2	18678.1	17633.3	26126.2	11802.3	10313.1	9136.9	7716.7	17421.6	13633.6	16363.5	13764.2	12483.8	9568.3	12184.9	12211.7	7756.3	10712.1	17119.7	21955.9	15490.4	17688.1	8832.9	12303.0	10405.7	hsa-mir-30a
hsa-miR-30a-5p	49940.7	32363.5	58270.5	51403.9	56846.0	44796.9	48889.9	59147.9	52602.8	48895.0	42878.9	41259.3	52222.5	55565.8	49147.1	50582.2	58961.6	38858.6	35941.5	48798.1	10582.2	40495.4	47936.6	56080.5	34910.3	49746.2	42345.6	46136.3	41566.0	hsa-mir-30a

Table S2

hsa-miR-30c-5p	685.5	909.7	555.6	197.3	385.8	621.2	308.9	507.8	451.0	307.8	97.0	70.1	374.6	304.9	627.9	253.4	225.4	289.9	235.6	399.2	1005.8	436.9	431.4	641.8	445.3	518.6	298.2	365.7	299.3	hsa-mir-30c-1
hsa-miR-30c-2-3p	734.7	530.7	724.7	498.6	174.4	308.7	272.3	476.6	150.6	451.2	452.8	273.3	382.6	221.9	307.7	222.7	250.5	85.2	207.9	604.4	241.2	487.4	211.6	310.1	347.8	210.4	110.2	237.3	174.5	hsa-mir-30c-2
hsa-miR-30c-5p	685.5	909.7	555.6	197.3	385.8	621.2	308.9	507.8	451.0	307.8	97.0	70.1	374.6	304.9	627.9	253.4	225.4	289.9	235.6	399.2	1005.8	436.9	431.4	641.8	445.3	518.6	298.2	365.7	299.3	hsa-mir-30c-2
hsa-miR-30d-5p	5135.4	6379.9	4050.3	2957.5	3196.3	4974.0	3280.9	4073.8	3899.6	3987.1	2581.3	1598.3	4945.7	3930.5	4484.3	3387.9	2151.6	4295.2	1958.9	4348.3	5902.0	3863.6	4086.4	4654.0	4936.9	3974.0	3027.6	2776.7	3719.8	hsa-mir-30d
hsa-miR-30e-5p	1076.3	932.7	826.5	912.6	677.0	771.0	599.6	514.3	740.6	951.2	607.5	412.6	565.7	923.6	465.0	764.5	676.1	550.5	383.5	872.4	417.8	769.4	523.6	800.4	862.8	869.3	763.4	531.6	551.4	hsa-mir-30e
hsa-miR-30e-3p	1674.9	2171.1	1497.5	1879.1	2127.0	2891.4	2304.5	3183.7	1831.8	1658.4	1360.5	1091.7	2311.2	2280.7	1897.9	2248.9	1566.5	2064.8	1746.1	1855.4	1984.1	1584.2	2863.5	3241.4	3144.1	2320.5	1459.0	2302.0	1655.8	hsa-mir-30e
hsa-miR-320a	5998.0	6912.2	3887.4	2226.5	2494.1	4528.0	2935.7	5534.4	2412.4	2408.8	2756.6	1513.9	3336.7	2777.0	4018.1	2262.4	4533.6	3432.5	2981.4	3680.0	5938.1	3548.9	2816.4	3832.8	4087.3	3814.4	3535.9	2599.5	2468.2	hsa-mir-320a
hsa-miR-328-3p	190.3	295.2	102.8	70.0	69.2	141.3	142.3	89.5	138.6	52.4	35.2	55.7	70.0	41.1	81.4	43.3	86.5	108.8	45.1	168.0	92.6	183.1	119.8	104.7	121.4	114.0	41.7	126.2	46.6	hsa-mir-328
hsa-miR-375	9697.2	15119.7	189.9	1373.0	962.5	1847.9	2132.5	5413.4	1650.4	956.0	1763.0	971.3	12384.0	1363.1	4181.4	3037.9	614.1	3875.1	4156.3	1249.1	21208.7	3742.0	972.7	4343.1	6070.8	2075.1	508.6	3292.7	6037.6	hsa-mir-375
hsa-miR-378a-3p	1176.5	1500.3	1370.6	533.4	689.7	1126.2	1127.1	1294.8	677.4	538.9	634.5	406.7	1078.8	759.7	830.1	917.5	1545.9	847.5	755.1	446.8	562.9	727.5	1116.6	639.8	1034.8	627.1	1409.4	424.2	611.0	hsa-mir-378a
hsa-miR-423-3p	483.6	751.6	438.3	216.0	229.2	379.7	327.3	395.1	164.7	367.9	282.6	168.9	165.7	205.8	119.2	87.2	393.2	217.0	139.3	427.7	613.9	434.5	203.7	160.6	256.3	180.2	168.0	181.5	137.9	hsa-mir-423
hsa-miR-423-5p	2237.0	1889.5	2070.1	1511.1	1234.0	1824.8	1369.3	1333.0	1515.0	1886.2	1500.9	1194.5	1015.8	1502.4	1207.0	1052.7	1057.7	1178.2	822.8	1843.4	1244.7	1757.3	1185.8	1210.6	1514.7	1152.9	1087.0	858.6	1008.2	hsa-mir-423
hsa-miR-486-5p	1321.0	768.2	1696.4	2528.9	394.8	424.7	569.1	793.6	195.7	2038.2	1293.4	1130.9	272.9	1017.1	290.0	3114.7	428.1	429.1	772.8	1210.0	3031.8	2324.9	334.8	233.1	603.7	298.1	1606.3	319.0	597.7	hsa-mir-486-1
hsa-miR-486-5p	1325.1	774.4	1686.8	2493.7	394.8	424.9	569.1	793.6	196.0	2032.5	1307.4	1127.2	256.9	1017.6	290.0	3108.6	428.1	429.1	772.8	1217.6	3060.9	2324.9	325.4	228.8	604.1	294.4	1591.1	319.0	581.0	hsa-mir-486-2
hsa-miR-532-5p	664.0	944.0	523.5	337.6	110.4	110.5	71.7	183.3	103.2	469.0	708.4	311.3	209.2	129.6	98.8	108.1	45.9	31.6	31.4	772.4	714.3	418.4	96.1	118.4	71.1	127.2	51.7	44.0	132.5	hsa-mir-532
hsa-miR-574-5p	319.0	596.0	137.2	109.3	102.7	405.9	174.3	220.6	73.1	107.8	109.2	39.7	290.3	92.7	176.7	132.3	191.8	205.7	172.3	146.3	1027.0	245.1	123.7	212.2	449.1	143.5	141.9	174.5	159.2	hsa-mir-574
hsa-miR-577	144.5	145.3	101.2	141.9	109.1	147.8	185.4	263.5	126.6	258.3	263.9	181.5	44.6	316.3	23.2	172.7	140.8	121.7	53.3	180.5	31.4	129.8	96.4	51.9	131.8	86.9	96.9	54.2	80.9	hsa-mir-577
hsa-miR-660-5p	104.0	114.8	134.3	53.2	61.2	146.4	59.5	119.3	109.4	118.8	77.6	31.2	119.4	87.2	130.6	94.0	113.0	80.4	23.1	94.5	58.8	80.0	66.7	93.6	211.9	127.2	67.3	81.1	83.9	hsa-mir-660
hsa-miR-769-5p	112.0	142.9	100.9	138.9	125.9	72.9	149.1	222.3	91.3	100.7	95.9	69.2	229.8	174.0	149.5	101.4	164.0	202.1	46.3	94.0	236.1	88.3	101.3	118.7	121.7	83.9	59.6	43.5	84.6	hsa-mir-769
hsa-miR-9-5p	117.8	168.0	60.4	391.2	300.9	112.2	166.7	588.9	186.3	239.1	375.6	295.8	159.1	254.5	469.8	176.8	296.4	324.2	162.8	181.0	41.6	296.6	145.6	372.0	386.3	363.6	194.5	533.8	80.6	hsa-mir-9-1
hsa-miR-9-5p	117.8	168.0	60.4	391.2	300.9	112.2	166.7	588.9	186.3	239.1	375.6	295.8	159.1	254.5	469.8	176.8	296.4	324.2	162.8	181.0	41.6	296.6	145.6	372.0	386.3	363.6	194.5	533.8	80.6	hsa-mir-9-2
hsa-miR-92a-3p	18245.8	26609.6	14400.4	15437.1	1452.2	3132.7	1865.9	2337.5	1184.5	11876.4	16361.3	7054.8	4185.1	2184.2	2314.2	1926.5	813.0	904.7	815.4	13259.8	28994.4	13359.0	1730.0	2754.2	2615.0	1340.2	1059.7	718.5	1468.4	hsa-mir-92a-1
hsa-miR-92a-3p	18857.4	27672.0	14700.8	15841.7	1528.9	3290.2	1914.3	2656.3	1267.0	12136.2	16569.2	7085.7	4459.8	2327.3	2431.4	2033.5	813.0	950.9	818.3	13487.9	30830.6	13833.2	1831.1	2864.0	2853.9	1417.1	1106.1	739.4	1618.2	hsa-mir-92a-2
hsa-miR-92b-3p	555.2	544.7	547.6	174.4	456.2	421.0	578.6	570.5	633.8	595.5	322.1	272.4	288.6	561.5	664.9	553.3	223.4	639.3	140.5	772.0	22.4	388.8	546.5	671.8	378.3	600.9	470.6	178.3	509.4	hsa-mir-92b
hsa-miR-93-5p	190.7	529.7	170.0	147.5	65.7	107.7	247.5	197.3	109.1	71.7	88.3	51.8	239.2	80.5	155.4	130.2	98.8	135.0	47.9	108.4	700.6	140.8	117.5	145.2	153.3	148.6	212.9	79.5	87.2	hsa-mir-93
hsa-miR-9-5p	117.8	168.0	60.4	391.2	300.9	112.2	166.7	588.9	186.3	239.1	375.6	295.8	159.1	254.5	469.8	176.8	296.4	324.2	162.8	181.0	41.6	296.6	145.6	372.0	386.3	363.6	194.5	533.8	80.6	hsa-mir-9-3
hsa-miR-941	190.9	325.7	129.2	181.6	152.3	317.9	85.4	211.8	127.2	157.3	105.2	190.0	309.1	152.0	157.4	202.4	166.6	177.9	223.6	331.1	248.7	150.1	139.9	86.2	479.6	256.7	225.7	165.4	197.1	hsa-mir-941-1
hsa-miR-941	190.9	325.7	129.2	181.6	152.3	317.9	85.4	211.8	127.2	157.3	105.2	190.0	309.1	152.0	157.4	202.4	166.6	177.9	223.6	331.1	248.7	150.1	139.9	86.2	479.6	256.7	225.7	165.4	197.1	hsa-mir-941-2
hsa-miR-941	190.9	325.7	129.2	181.6	152.3	317.9	85.4	211.8	127.2	157.3	105.2	190.0	309.1	152.0	157.4	202.4	166.6	177.9	223.6	331.1	248.7	150.1	139.9	86.2	479.6	256.7	225.7	165.4	197.1	hsa-mir-941-3
hsa-miR-941	190.9	325.7	129.2	181.6	152.3	317.9	85.4	211.8	127.2	157.3	105.2	190.0	309.1	152.0	157.4	202.4	166.6	177.9	223.6	331.1	248.7	150.1	139.9	86.2	479.6	256.7	225.7	165.4	197.1	hsa-mir-941-4
hsa-miR-941	190.9	325.7	129.2	181.6	152.3	317.9	85.4	211.8	127.2	157.3	105.2	190.0	309.1	152.0	157.4	202.4	166.6	177.9	223.6	331.1	248.7	150.1	139.9	86.2	479.6	256.7	225.7	165.4	197.1	hsa-mir-941-5
hsa-miR-98-5p	979.6	1729.0	702.5	565.6	551.8	1290.5	916.9	1214.1	448.7	408.8	450.3	297.2	640.2	439.1	771.1	578.9	324.2	661.6	564.1	484.5	1793.1	820.1	833.5	715.7	1597.9	840.5	643.9	474.1	736.5	hsa-mir-98
hsa-miR-99a-5p	472.9	396.4	292.4	271.4	209.2	221.3	116.0	360.9	207.1	359.1	398.2	266.5	222.8	227.0	303.3	223.0	332.6	281.5	145.5	325.0	448.0	495.8	251.7	214.8	143.6	301.8	176.1	249.2	160.5	hsa-mir-99a
hsa-miR-99b-5p	4929.5	2946.3	5716.2	3831.6	3525.3	3780.9	4040.7	2860.6	2756.9	5419.5	4861.1	4259.1	2612.3	3957.2	3668.7	2886.4	4189.5	3296.5	2839.6	5362.5	817.5	4631.6	3279.5	3233.1	3247.8	2969.5	5258.1	2955.5	3125.8	hsa-mir-99b
hsa-miR-148b-3p	229.4	366.4	105.1	289.4	56.3	160.9	98.4	288.1	38.0	137.8	345.8	126.4	136.5	81.7	57.4	64.5	0.0	43.6	59.5	147.3	833.6	374.2	129.0	150.1	157.1	67.0	75.0	133.7	101.2	hsa-mir-148b
hsa-miR-31-5p	69.2	163.1	74.2	6.0	38.2	105.7	268.5	184.2	92.4	119.4	104.5	82.8	129.2	135.5	298.2	124.0	54.2	220.5	186.8	113.3	53.0	109.9	100.8	207.1	1035.8	188.0	781.8	76.8	96.6	hsa-mir-31
hsa-miR-425-5p	102.8	127.1	96.1	32.9	9.5	60.4	72.1	104.4	72.8	22.8	74.7	39.7	77.6	45.7	42.5	19.7	118.2	80.7	17.8	18.9	67.9	76.1	79.9	88.2	95.7	35.3	166.1	56.4	84.2	hsa-mir-425
hsa-miR-140-3p	260.1	403.6	325.5	207.0	68.1	115.6	8.4	163.6	25.2	155.2	195.7	93.2	125.3	100.4	168.4	58.9	41.3	21.6	21.5	189.0	576.6	130.0	34.6	100.4	71.8	54.6	42.9	70.3	6.3	hsa-mir-140
hsa-miR-151a-5p	61.8	63.9	50.1	0.0	28.6	164.1	77.0	139.0	62.0	35.9	50.6	19.1	183.1	14.4	48.0	40.7	0.0	59.4	45.9	87.2	63.9	74.2	48.6	106.7	115.1	45.6	79.9	24.2	38.3	hsa-mir-151a
hsa-miR-151b	61.8	63.9	50.1	0.0	28.6	164.1	77.0	139.0	62.0	35.9	50.6	19.1	183.1	14.4	48.0	40.7	0.0	59.4	45.9	87.2	63.9	74.2	48.6	106.7	115.1	45.6	79.9	24.2	38.3	hsa-mir-151b
hsa-miR-152-3p	83.3	79.8	68.5	46.8	40.0	40.2	21.4	79.4	88.6	68.7	29.8	0.0	28.9	33.0	151.1	68.1	0.0	91.1	74.4	106.5	120.4	80.2	58.5	49.6	47.2	78.8	107.6	69.3	47.3	hsa-mir-152
hsa-miR-23b-3p	35.5	127.9	27.3	20.2	35.2	132.7	67.1	230.2	32.2	12.7	27.7	14.4	71.0	36.8	84.2	88.7	0.7	80.1	51.7	41.2	213.0	40.7	66.0	102.7	112.7	89.2	20.7	8.6	22.0	hsa-mir-23b
hsa-miR-24-3p	71.9	69.1	81.3	0.0	23.2	17.1	42.7	69.3	33.3	8.3	58.9	12.4	44.9	86.8	95.2	38.3	56.8	14.2	43.8	33.0	53.0	56.7	50.8	18.3	21.9	29.3	73.6	15.6	66.3	hsa-mir-24-1
hsa-miR-24-3p	71.9	69.1	81.3	0.0	23.4	17.1	42.7	69.3	33.3	8.3	58.9	12.4	44.9	86.8	95.2	38.3	56.8	14.2	43.8	33.0	53.0	56.7	50.8	18.3	21.9	29.3	73.6	15.6	66.3	hsa-mir-24-2
hsa-miR-421	23.3	79.6	28.6	21.0	67.0	31.3	101.5	142.5	41.8	30.8	36.3	0.0	25.1	46.6	33.4	35.4	35.5	14.2	69.4	20.7	33.7	39.5	27.6	14.0	0.0	34.3	24.9	72.5	14.0	hsa-mir-421
hsa-miR-744-5p	41.7	83.8	73.9	24.3	19.6	71.8	99.6	46.9	29.3	14.8	0.0	21.4	43.9	73.3	29.9	18.3	42.0	28.7	34.7	14.9	158.5	46.2	19.2	44.2	60.0	46.5	24.2	0.0	29.6	hsa-mir-744
hsa-miR-16-5p	31.9	42.5	62.7	1.1	14.6	56.1	1.9	0.9	103.0	19.6	13.6	25.9	36.9	39.4	52.3	35.7	41.3	72.7	35.5	50.2	84.0	10.1	56.0	33.4	164.0	33.0	154.7	91.8	24.0	hsa-mir-16-1
hsa-miR-16-5p	31.9	42.5	62.7	1.1	14.6	56.1	1.9	0.9	103.0	19.6	13.6	25.9	36.9	39.4	52.3	35.7	41.3	72.7	35.5	50.2	84.3	10.1	56.0	33.4	164.0	33.0	154.7	91.8	24.0	hsa-mir-16-2
hsa-miR-196b-5p	244.9	359.4	178.4	60.6	56.9	48.7	0.0	56.1	36.0	51.0	50.3	10.1	78.3	0.4	119.6	47.7	87.8	54.2	18.2	56.8	831.2	139.6	0.0	71.9	154.7	27.9	54.1	46.7	49.6	hsa-mir-196b
hsa-miR-363-3p	140.5	206.8	46.9	46.4	41.7	91.7	24.4	94.3	60.8	35.9	11.9	14.4	109.0	0.0	55.1	43.9	0.0	39.7	0.0	74.9	375.8	71.6	36.6	53.6	128.3	32.7	36.8	47.3	71.9	hsa-mir-363
hsa-miR-574-3p	105.8	259.1	54.0	0.0	47.5	259.2	140.7	46.0	19.3	32.6	13.6	14.6	101.3	71.6	99.1	47.4	0.7	39.4	79.4	42.4	972.8	61.5	2.5	31.7	128.3	91.0	94.6	72.5	31.0	hsa-mir-574
hsa-miR-891a-5p	153.2	311.3	0.3	422.6	230.9	243.0	32.8	355.2	208.0	177.5	116.7	37.2	41.4	184.2	22.4	73.4	53.0	1281.8	0.0	202.9	0.0	52.2	264.1	102.1	185.5	201.8	117.9	39.2	510.1	hsa-mir-891a
hsa-miR-99b-3p	65.6	71.6	53.3	31.4	18.7	157.5	53.0	92.5	34.2	40.6	35.6	7.9	88.4	72.0	60.2	69.2	0.0	88.5	85.1	45.2	94.9	57.7	42.3	88.4	100.6	110.8	55.9	0.0	49.6	hsa-mir-99b
hsa-miR-1180-3p	103.2	68.3	108.3	0.0	30.9	78.6	64.1	68.8	52.9	0.0	10.4	29.0	34.1	14.4	17.7	24.2	0.0	32.6	35.5	34.4	62.8	63.0	0.5	42.8	78.7	75.6	51.5	77.9	24.6	hsa-mir-1180
hsa-miR-30b-5p	40.3	63.5	31.8	6.7	32.4	108.8	45.4	42.5	23.4	14.5	25.9	16.6	21.6	28.0	36.6	23.0	8.4	20.7	0.0	15.6	91.0	43.3	51.0	68.2	22.2	32.7	38.7	0.0	10.7	hsa-mir-30b
hsa-miR-30b-3p	17.3	43.7	16.7	12.4	34.6	53.8	70.2	97.3	59.7	16.3	24.1	0.0	17.8	16.9	12.2	53.0	0.7	42.0	46.3	70.5	29.4	6.9	13.2	45.4	74.6	49.1	20.7	0.0	37.6	hsa-mir-30b
hsa-miR-4446-3p	53.4	41.1	15.8	17.6	21.5	25.1	111.8	103.9	22.2	50.1	12.6	15.2	0.0	53.8	8.3	27.1	34.9	37.1	0.0	50.4	22.8	54.8	23.4	19.4	293.4	1.6	33.6	24.7	23.0	hsa-mir-4446
hsa-miR-340-5p	47.4	60.3	16.1	15.0	14.6	38.7	21.4	26.8	0.0	19.0	95.9	40.5	34.5	18.6	22.4	15.6	0.0	0.0	0.0	39.6	42.0	38.3	22.4	52.2	22.2	33.6	17.7	0.0	19.3	hsa-mir-340
hsa-miR-598-3p	66.4	77.2	39.2	27.3	15.0	69.5	29.4	38.6	0.0	14.8	0.0	17.7	45.6	0.0	44.1	14.1	23.9	14.9	0.0	19.1	72.2	58.7	41.1	38.8	21.2	27.9	19.1	94.5	9.3	hsa-mir-598
hsa-miR-210-3p	61.2	55.5	41.8	16.1	8.4	74.1	0.0	20.2	11.7	37.6	58.2	42.2	15.0	28.0	66.1	15.0	0.0	56.5	0.0	49.5	37.3	45.2	0.5	55.3	68.7	39.9	45.4	0.0	19.6	hsa-mir-210
hsa-miR-28-5p	51.0	59.9	8.4	14.2	19.8	80.6	98.8	15.8	14.0	45.0	15.4	0.0	0.4	19.1	46.8	0.0	43.3	13.6	0.0	13.7	150.2	35.2	14.9	63.0	23.9	35.0	26.8	0.0	11.3	hsa-mir-28
hsa-miR-3615	25.3	53.9	58.8	41.2	0.0	29.6	70.6	92.5	31.3	0.0	38.1	11.8	7.0	30.5	107.0	11.8	0.0	60.7	30.2	47.8	22.4	7.9	22.7	23.4	91.6	41.2	33.6	98.3	9.7	hsa-mir-3615
hsa-miR-374a-3p	33.5	21.0	10.6	11.6	50.3	0.0	63.3	33.3	36.6	34.4	27.7	0.0	8.4	46.2	64.1	43.9	0.0	100.1	0.0	23.3	25.1	34.5	24.4	46.8	25.0	34.3	20.7	0.5	13.0	hsa-mir-374a
hsa-miR-425-3p	18.3	47.5	28.0	0.0	44.5	32.2	17.2	27.2	13.2	11.3	28.7	15.8	25.8	0.0	0.0	13.3	0.0	40.4	19.8	48.1	40.0	45.2	13.9	24.8	0.0	17.1	22.1	0.0	12.3	hsa-mir-425
hsa-miR-7706	7.6	32.1	49.2	7.9	44.5	17.9	0.0	36.0	32.5	20.7	29.4	42.5	5.9	32.2	17.3	0.0	46.5	12.0	87.6	39.4	3.9	30.2	51.8	14.0	87.7	26.3	77.6	24.2	27.3	hsa-mir-7706

hsa-miR-130a-3p	56.2	49.7	103.2	0.0	83.6	13.1	33.2	0.0	36.9	50.1	29.4	8.7	42.1	42.4	0.0	17.1	0.0	24.5	24.0	27.8	0.0	16.8	4.0	16.8	46.5	29.0	31.2	27.4	47.3	hsa-mir-130a
hsa-miR-184	152.0	62.3	168.4	110.4	31.1	52.4	16.8	56.6	0.0	80.3	27.7	92.0	19.2	59.3	27.5	43.3	0.0	25.8	0.0	32.8	9.8	52.4	53.8	0.0	19.1	7.8	34.7	69.8	3.0	hsa-mir-184
hsa-miR-19b-3p	27.5	72.2	7.4	34.1	31.4	36.2	29.4	25.0	22.2	10.4	40.6	12.7	45.6	0.0	50.4	29.5	0.0	36.5	20.7	32.8	125.1	6.0	0.0	9.1	14.6	22.6	33.1	43.0	8.7	hsa-mir-19b-1
hsa-miR-19b-3p	27.5	72.2	7.4	34.1	31.4	36.2	29.4	25.0	22.2	10.4	40.6	12.7	45.6	0.0	50.4	29.5	0.0	36.5	20.7	32.8	125.1	6.0	0.0	9.1	14.6	22.6	33.1	43.0	8.7	hsa-mir-19b-2
hsa-miR-429	35.3	141.5	26.7	32.9	9.0	37.0	19.8	133.3	14.0	67.3	0.0	23.6	0.0	22.0	33.8	11.8	0.0	73.9	0.0	0.0	138.1	40.9	26.6	40.2	81.5	48.8	51.7	0.0	19.3	hsa-mir-429
hsa-miR-92b-5p	15.3	25.1	18.0	0.0	23.2	20.2	0.0	20.2	11.4	37.9	33.0	6.2	11.8	14.4	0.4	15.3	82.0	25.2	0.0	9.0	3.1	14.8	14.2	19.7	69.0	34.6	21.9	25.2	42.0	hsa-mir-92b
hsa-miR-342-3p	39.1	52.1	64.3	18.0	45.3	22.8	0.0	0.0	25.5	37.9	107.7	38.3	23.3	0.0	27.5	0.3	0.0	0.0	21.9	31.6	64.7	20.4	51.8	21.7	24.6	9.5	8.9	25.2	13.0	hsa-mir-342
hsa-miR-500a-3p	138.9	204.0	207.0	102.2	15.0	10.8	16.0	0.0	0.0	200.9	265.7	252.2	14.3	15.7	40.9	27.1	0.0	0.3	18.6	184.5	82.4	201.6	0.0	21.1	15.6	17.7	0.0	0.5	7.0	hsa-mir-500a
hsa-miR-181a-3p	14.8	37.5	30.9	21.3	9.2	15.7	0.0	0.0	12.3	21.6	23.3	15.2	0.0	25.0	55.1	12.4	0.0	20.7	41.3	0.0	11.4	27.1	31.6	11.1	3.5	9.2	31.5	20.4	8.7	hsa-mir-181a-1
hsa-miR-320b	28.1	43.9	30.5	35.9	30.1	13.4	38.1	28.9	2.3	2.1	18.7	26.2	2.8	2.5	17.3	3.0	7.1	21.6	28.1	17.2	24.7	12.5	3.5	28.8	57.9	16.6	12.8	3.8	5.0	hsa-mir-320b-2
hsa-miR-361-5p	30.9	50.9	18.0	19.1	20.6	16.2	0.0	75.4	40.4	0.0	0.0	0.0	15.0	8.9	27.5	0.0	0.0	54.6	19.4	5.4	10.6	31.1	13.7	21.7	70.7	16.6	73.6	0.0	39.6	hsa-mir-361
hsa-miR-424-3p	28.3	37.5	66.2	10.9	45.1	71.5	38.9	81.6	0.0	13.0	24.4	0.0	0.0	13.1	0.0	0.3	22.0	58.4	34.7	0.0	4.7	18.9	58.0	39.1	20.5	11.1	0.0	0.0	43.6	hsa-mir-424
hsa-miR-92a-1-5p	45.4	73.8	18.3	37.8	27.7	81.7	0.0	44.7	34.2	0.0	0.0	13.2	75.2	13.1	2.8	26.8	0.0	16.8	20.7	0.0	106.3	6.9	0.3	30.2	26.4	17.5	0.0	24.7	63.6	hsa-mir-92a-1
hsa-miR-320b	28.1	62.3	30.5	35.9	30.1	3.4	37.8	28.5	2.3	2.1	34.1	26.2	3.1	2.5	17.3	2.7	7.1	21.0	27.7	17.4	32.2	2.9	3.5	28.8	30.9	20.3	15.4	3.8	15.0	hsa-mir-320b-1
hsa-miR-345-5p	69.2	83.6	26.0	0.0	24.1	28.2	2.7	0.0	0.0	70.2	48.1	11.5	55.4	12.3	8.7	0.0	0.0	0.0	0.0	65.3	96.9	26.8	30.9	14.8	40.9	25.1	18.6	0.0	12.7	hsa-mir-345
hsa-miR-584-5p	25.9	110.6	53.7	16.8	4.9	41.6	117.9	32.0	0.0	40.3	27.7	0.0	16.7	0.4	34.2	35.7	0.0	0.0	19.8	10.6	4.3	69.7	12.7	1.7	45.4	18.2	20.3	0.0	8.0	hsa-mir-584
hsa-miR-671-3p	55.4	45.9	0.0	0.0	0.0	17.4	38.9	21.1	5.6	32.0	26.2	0.0	11.8	16.1	9.8	0.0	40.7	4.8	19.4	15.1	85.9	46.0	20.9	10.0	19.8	15.2	24.9	0.0	23.6	hsa-mir-671
hsa-miR-1226-5p	22.5	33.7	10.6	71.9	12.5	0.0	19.8	0.0	0.0	9.8	22.6	22.2	0.0	25.4	13.0	14.4	0.0	13.9	0.0	9.4	16.5	19.6	23.2	0.0	34.7	0.0	15.1	0.0	33.3	hsa-mir-1226
hsa-miR-1270	16.5	17.6	0.0	0.0	17.2	38.7	0.0	28.9	0.0	0.0	0.0	25.9	23.7	35.2	9.4	31.2	42.6	14.2	0.0	23.8	34.1	14.1	6.0	0.0	66.9	44.9	7.2	14.0	19.6	hsa-mir-1270
hsa-miR-218-5p	10.6	13.8	6.4	44.2	12.7	38.2	0.0	20.2	9.1	20.1	12.2	0.0	0.0	13.1	15.3	9.7	0.0	14.9	0.0	13.7	11.8	0.0	48.6	22.8	0.0	15.9	12.8	0.0	19.0	hsa-mir-218-1
hsa-miR-218-5p	10.6	13.8	6.4	44.2	12.7	38.2	0.0	20.2	9.1	20.1	12.2	0.0	0.0	13.1	15.3	9.7	0.0	14.9	0.0	13.7	11.8	0.0	48.6	22.8	0.0	15.9	12.8	0.0	19.0	hsa-mir-218-2
hsa-miR-29a-3p	15.1	34.9	8.4	0.0	12.0	39.9	24.0	31.1	0.0	19.6	15.4	24.5	0.0	23.7	0.0	0.3	0.0	12.9	0.0	44.5	43.2	9.6	0.3	28.5	48.6	16.1	19.8	0.5	16.0	hsa-mir-29a
hsa-miR-342-5p	41.9	64.9	51.4	32.2	0.0	49.8	0.0	0.0	30.1	0.0	29.1	0.0	0.0	31.3	0.0	18.6	0.0	24.2	0.0	0.0	35.7	26.6	10.5	20.8	16.0	16.4	12.4	39.7	5.0	hsa-mir-342
hsa-miR-1468-5p	23.9	17.2	11.9	16.8	0.0	0.0	0.0	0.0	0.0	0.3	11.9	11.5	21.2	34.7	14.6	3.0	0.0	11.3	0.0	22.2	18.8	23.2	0.3	28.5	20.5	13.4	0.0	0.0	10.3	hsa-mir-1468
hsa-miR-29c-3p	22.1	23.5	11.9	18.3	2.2	28.8	22.1	11.0	24.6	8.9	0.0	14.4	14.6	12.7	0.0	12.4	0.0	0.0	0.0	16.0	7.9	28.7	0.0	0.6	44.4	5.1	21.0	0.0	0.0	hsa-mir-29c
hsa-miR-324-5p	19.3	28.9	0.0	0.0	0.0	23.4	0.0	0.0	0.0	18.1	13.6	0.0	43.5	0.0	11.0	13.0	0.0	11.0	12.8	0.0	44.7	16.0	0.0	33.4	21.9	15.2	26.1	0.0	0.0	hsa-mir-324
hsa-miR-365b-5p	16.9	27.3	25.4	15.4	10.7	30.2	29.4	18.0	0.0	0.0	13.3	13.0	11.1	5.1	0.0	24.5	0.0	0.0	0.0	6.6	4.3	43.6	0.0	0.0	18.4	10.8	0.0	4.8	33.3	hsa-mir-365b
hsa-miR-432-5p	9.0	101.4	100.3	0.0	0.0	72.6	42.3	63.1	10.8	0.0	0.0	34.6	27.9	0.0	0.0	34.2	54.2	0.0	2.1	15.3	47.5	17.0	14.7	9.4	27.4	2.5	6.3	52.1	0.0	hsa-mir-432
hsa-miR-99a-3p	19.9	26.9	27.0	0.0	0.0	8.5	28.6	21.5	0.0	20.1	20.8	0.0	17.8	14.4	0.0	0.0	0.0	20.0	22.7	22.2	52.6	22.0	13.7	0.0	0.0	12.9	0.0	0.0	0.0	hsa-mir-99a
hsa-miR-10b-3p	32.3	8.2	19.0	40.8	18.3	19.9	18.3	0.0	58.8	14.8	13.6	0.0	0.0	0.4	15.0	30.9	0.0	13.2	0.0	11.1	3.1	8.6	0.0	0.0	0.0	2.8	19.3	0.0	52.6	hsa-mir-10b
hsa-miR-194-5p	49.4	33.5	29.6	0.0	0.0	20.8	0.0	57.4	24.9	13.9	19.0	0.0	0.0	0.0	30.7	29.5	0.0	11.0	0.0	0.5	10.2	6.9	28.6	10.8	0.0	6.9	16.5	0.0	0.0	hsa-mir-194-2
hsa-miR-197-3p	15.3	36.3	14.5	0.0	6.4	17.4	18.3	14.0	9.4	25.8	11.1	0.0	32.7	0.0	0.0	0.0	0.0	0.0	0.0	0.0	25.9	0.2	34.4	24.0	20.5	13.6	18.9	0.0	0.0	hsa-mir-197
hsa-miR-132-3p	23.9	58.5	17.0	17.6	0.0	0.0	0.0	25.9	0.0	7.4	12.6	0.0	22.3	0.0	13.8	0.0	0.7	12.9	64.1	0.0	74.1	0.0	0.0	26.2	0.0	10.1	14.0	0.0	0.7	hsa-mir-132
hsa-miR-146b-3p	7.6	10.0	0.0	0.0	0.0	33.3	20.6	0.0	0.0	9.8	0.0	0.0	15.0	11.0	18.1	15.3	49.1	0.0	15.7	15.8	9.0	11.0	14.7	0.0	0.0	6.5	17.0	24.7	0.0	hsa-mir-146b
hsa-miR-194-5p	49.2	33.3	28.9	0.0	0.0	20.8	0.0	57.4	24.9	25.5	28.0	0.0	0.0	0.0	30.7	18.6	0.0	11.0	0.0	0.5	3.5	6.9	28.6	10.8	0.0	6.9	16.5	0.0	0.0	hsa-mir-194-1
hsa-miR-365a-5p	19.1	14.6	0.0	18.7	20.2	0.0	19.5	69.7	0.0	0.0	0.0	0.0	11.8	0.0	11.4	0.0	0.0	11.6	0.0	6.8	0.0	7.4	20.9	25.4	26.0	2.3	12.8	0.0	10.7	hsa-mir-365a
hsa-miR-484	27.5	28.9	15.1	0.0	15.5	17.7	0.0	37.3	9.9	9.8	12.9	13.2	0.0	0.0	12.6	13.3	0.0	41.7	0.0	0.0	28.2	6.5	0.0	0.0	0.0	8.1	22.8	0.0	11.0	hsa-mir-484
hsa-miR-128-3p	6.6	5.8	11.6	13.1	27.1	13.4	0.0	68.4	5.6	0.0	0.0	31.8	23.3	36.0	0.0	0.0	0.0	0.0	0.0	0.0	39.2	4.8	13.7	0.0	23.6	15.4	0.0	30.6	0.0	hsa-mir-128-1
hsa-miR-27b-5p	21.1	17.2	0.0	0.0	24.5	0.0	0.0	0.0	0.0	12.2	16.2	0.0	12.5	0.0	18.9	10.3	0.0	28.4	0.0	17.2	16.9	0.0	14.4	0.0	0.0	13.4	2.1	0.0	6.0	hsa-mir-27b
hsa-miR-589-5p	13.8	25.5	9.0	20.2	0.0	0.0	6.1	19.7	0.0	19.9	14.4	25.3	0.0	18.6	0.0	0.0	0.0	0.0	0.0	25.0	21.2	48.4	0.0	11.7	0.0	9.0	14.0	0.0	9.7	hsa-mir-589
hsa-miR-125b-5p	9.6	10.6	6.8	0.0	17.2	0.3	0.0	40.3	9.4	0.0	0.0	9.6	0.4	19.9	9.8	11.8	0.0	13.2	15.3	0.0	5.1	7.9	35.4	15.4	0.4	11.3	10.0	0.0	13.0	hsa-mir-125b-1
hsa-miR-125b-5p	9.6	10.6	6.8	0.0	17.2	0.3	0.0	40.3	9.4	0.0	0.0	9.6	0.4	19.9	9.8	11.8	0.0	13.2	15.3	0.0	5.1	7.9	35.4	15.4	0.4	11.3	10.0	0.0	13.0	hsa-mir-125b-2
hsa-miR-128-3p	6.6	5.8	11.6	0.0	40.0	13.4	0.0	68.4	0.0	0.0	0.0	31.8	11.5	36.0	0.0	0.0	0.0	0.0	0.0	8.0	38.8	4.8	13.4	0.0	23.6	15.4	0.0	18.3	0.0	hsa-mir-128-2
hsa-miR-181c-3p	0.2	5.0	0.0	18.0	14.0	20.5	0.0	0.0	0.0	0.0	32.3	0.0	11.5	14.0	28.7	40.4	0.0	0.0	0.0	0.0	8.2	16.8	0.0	14.0	4.2	5.5	27.5	27.9	4.7	hsa-mir-181c
hsa-miR-30d-3p	24.7	37.5	24.4	3.7	3.7	23.1	3.8	7.5	2.9	19.0	2.2	1.1	28.9	23.7	16.9	3.2	5.2	29.1	3.3	5.0	33.0	2.9	5.2	6.0	48.2	10.6	3.3	4.3	2.7	hsa-mir-30d
hsa-miR-361-3p	28.5	44.1	15.4	16.8	0.0	0.0	19.8	37.3	14.6	18.4	0.0	11.5	6.3	0.0	0.0	0.0	0.0	9.0	0.0	6.8	98.1	25.4	0.0	7.4	17.7	6.0	5.8	0.0	8.7	hsa-mir-361
hsa-miR-501-3p	35.3	11.0	46.6	110.8	10.1	0.0	0.0	25.0	0.0	45.3	34.8	81.3	17.4	0.0	3.5	0.0	0.0	0.0	0.0	8.7	0.4	46.4	13.7	0.3	0.0	0.0	0.0	0.0	0.0	hsa-mir-501
hsa-miR-874-3p	55.2	38.5	8.7	15.0	5.2	17.4	0.0	0.0	0.0	22.5	11.1	25.6	19.8	0.0	0.0	0.0	0.0	0.0	0.0	54.9	51.0	86.7	7.5	11.4	0.0	1.4	0.2	0.0	0.0	hsa-mir-874
hsa-miR-130b-3p	7.4	30.5	22.5	15.0	0.0	20.2	0.0	0.0	0.0	19.6	14.0	13.2	0.4	0.0	0.0	26.5	40.0	0.0	0.0	18.1	20.8	0.0	0.0	0.0	0.0	5.1	7.9	0.0	0.0	hsa-mir-130b
hsa-miR-200b-5p	13.8	10.8	7.7	14.6	0.0	0.0	0.0	0.0	13.2	0.0	12.9	12.7	0.0	13.6	0.0	0.0	0.0	0.0	0.0	18.1	40.4	0.0	0.0	9.4	17.3	2.5	7.0	0.0	13.3	hsa-mir-200b
hsa-miR-204-3p	24.1	20.0	5.5	16.5	8.2	0.6	0.0	0.0	19.3	24.9	24.1	0.0	0.0	17.4	21.6	0.0	0.0	0.0	0.0	9.4	0.0	20.6	13.0	8.8	0.7	11.3	0.0	0.0	0.0	hsa-mir-204
hsa-miR-409-3p	0.0	22.5	27.3	71.9	18.3	35.0	30.9	0.0	12.9	0.0	39.5	0.0	13.6	0.0	0.0	0.0	0.0	5.5	0.0	0.0	16.5	7.7	3.7	0.0	0.0	0.0	15.4	0.0	10.0	hsa-mir-409
hsa-miR-7-5p	12.0	36.1	20.9	19.1	0.0	5.1	0.0	84.6	0.0	8.3	0.0	34.1	0.0	16.5	0.0	0.0	0.0	25.2	0.0	9.2	119.6	9.6	0.0	8.6	32.9	9.7	26.6	0.0	0.7	hsa-mir-7-1
hsa-miR-7-5p	12.0	36.1	20.9	19.1	0.0	5.1	0.0	84.6	0.0	8.3	0.0	34.1	0.0	16.5	0.0	0.0	0.0	25.2	0.0	9.2	119.6	9.6	0.0	8.6	32.9	9.7	26.6	0.0	0.7	hsa-mir-7-2
hsa-miR-7-5p	15.0	36.1	20.9	19.1	0.0	5.1	0.0	84.6	0.0	8.3	0.0	34.1	0.0	16.5	0.0	0.0	0.0	25.5	0.0	9.2	119.6	9.6	0.0	8.6	32.9	9.7	26.6	0.0	0.7	hsa-mir-7-3
hsa-miR-873-3p	24.1	9.0	40.5	16.1	26.0	0.0	0.0	0.0	21.9	0.0	27.7	14.1	19.8	0.4	0.0	0.0	0.0	7.4	0.0	17.4	0.0	6.0	0.0	0.0	0.0	5.1	10.0	0.0	11.3	hsa-mir-873
hsa-let-7a-3p	10.6	31.5	18.3	0.0	0.0	0.0	21.7	23.2	0.0	0.0	18.3	0.0	5.2	14.8	0.0	0.0	0.0	16.5	8.3	9.2	68.3	10.1	0.0	0.0	0.0	6.9	0.0	0.0	0.0	hsa-let-7a-1
hsa-let-7a-3p	10.6	31.5	18.3	0.0	0.0	0.0	21.7	23.2	0.0	0.0	18.3	0.0	5.2	14.8	0.0	0.0	0.0	16.5	8.3	0.0	68.3	10.1	0.0	0.0	0.0	6.9	0.0	0.0	0.0	hsa-let-7a-3
hsa-miR-1262	12.8	11.2	15.8	7.5	0.0	0.0	15.6	18.0	0.0	8.6	0.0	0.0	0.0	0.0	13.4	0.0	0.0	0.0	0.0	0.0	19.2	15.8	0.0	10.3	45.8	2.5	8.2	0.0	6.0	hsa-mir-1262
hsa-miR-1269a	3.6	0.0	33.7	15.7	0.0	0.0	0.0	0.0	0.0	9.5	17.6	0.0	0.0	14.4	0.0	30.1	45.2	16.5	0.0	9.0	0.0	19.9	16.9	5.1	0.0	0.0	17.9	0.0	9.7	hsa-mir-1269a
hsa-miR-143-3p	8.2	0.6	34.1	83.5	29.0	2.3	0.0	89.9	52.9	18.7	1.4	18.0	0.4	13.1	0.8	0.3	3.2	27.4	0.4	7.1	2.4	1.9	0.3	0.6	3.1	0.2	12.8	3.2	8.0	hsa-mir-143
hsa-miR-3622a-5p	0.0	4.2	16.4	0.0	9.9	0.0	0.0	0.0	14.6	0.0	37.3	11.5	0.0	32.2	0.0	0.0	0.0	0.0	0.8	27.1	0.0	9.1	29.1	37.7	0.0	1.4	7.2	26.9	10.3	hsa-mir-3622a
hsa-miR-449c-5p	20.9	31.1	38.2	0.0	0.0	0.0	0.0	0.0	0.0	13.6	79.0	30.7	0.0	0.0	0.0	14.7	0.0	0.0	0.0	48.1	13.7	37.3	0.0	0.0	0.0	2.8	0.0	0.0	0.0	hsa-mir-449c
hsa-miR-92a-2-5p	43.2	36.3	0.0	0.0	0.0	0.0	16.4	0.0	18.1	0.0	21.5	0.0	39.7	8.1	26.0	0.0	0.0	0.0	14.1	0.0	42.8	8.1	14.2	0.0	0.0	3.5	5.8	0.0	7.0	hsa-mir-92a-2
hsa-miR-1307-5p	7.6	6.4	0.0	41.2	0.0	17.7	0.0	0.0	0.0	0.0	14.7	0.0	17.8	10.6	0.4	0.0	0.0	13.9	0.0	15.1	29.0	0.0	0.0	10.8	0.0	2.3	0.0	1.1	9.3	hsa-mir-1307

hsa-miR-17-5p	11.8	17.4	19.3	0.0	0.0	0.0	0.0	0.0	0.0	24.3	0.0	0.0	10.8	0.0	0.0	0.0	0.0	14.2	0.0	1.7	40.4	8.9	7.5	14.3	19.4	7.6	3.5	0.0	0.0	hsa-mir-17
hsa-miR-20b-5p	15.3	14.4	0.0	0.0	0.0	0.0	0.0	0.0	0.0	0.0	0.0	0.0	16.0	0.0	0.0	16.2	0.0	0.0	24.8	0.0	58.1	0.0	0.0	19.1	0.0	0.0	12.6	0.5	13.0	hsa-mir-20b
hsa-miR-30c-1-3p	9.4	11.2	18.0	0.8	15.9	0.3	0.4	0.0	9.4	9.5	0.0	0.0	40.0	0.0	12.2	0.0	0.0	10.0	0.0	1.2	6.3	7.9	4.2	11.4	0.0	9.5	4.2	14.0	21.0	hsa-mir-30c-1
hsa-miR-542-3p	17.7	0.0	24.1	0.0	24.1	0.0	0.0	0.0	0.0	0.3	0.0	0.0	0.0	0.0	11.4	28.9	0.0	0.0	0.0	8.7	0.0	0.0	13.7	11.7	23.6	0.0	19.8	0.0	0.0	hsa-mir-542
hsa-miR-642a-5p	5.4	4.8	12.9	19.8	0.0	0.0	0.0	3.5	18.7	0.6	12.6	0.0	12.2	61.4	0.0	0.0	0.0	12.6	0.0	0.0	5.5	0.0	2.0	0.0	0.0	6.2	11.2	20.9	9.3	hsa-mir-642a
hsa-miR-6859-5p	13.6	3.2	0.0	31.4	16.3	0.0	0.0	0.0	0.0	23.7	13.6	0.0	0.0	0.0	0.0	12.1	0.0	0.0	19.8	8.3	0.0	30.4	0.0	5.1	0.0	5.1	2.8	21.5	0.0	hsa-mir-6859-1
hsa-miR-6859-5p	13.6	3.2	0.0	31.4	16.3	0.0	0.0	0.0	0.0	23.7	13.6	0.0	0.0	0.0	0.0	12.1	0.0	0.0	19.8	8.3	0.0	30.4	0.0	5.1	0.0	5.1	2.8	21.5	0.0	hsa-mir-6859-2
hsa-miR-6859-5p	13.6	3.2	0.0	31.4	16.3	0.0	0.0	0.0	0.0	23.7	13.6	0.0	0.0	0.0	0.0	12.1	0.0	0.0	19.8	8.3	0.0	30.4	0.0	5.1	0.0	5.1	2.8	21.5	0.0	hsa-mir-6859-3
hsa-miR-6859-5p	13.6	3.2	0.0	31.4	16.3	0.0	0.0	0.0	0.0	23.7	13.6	0.0	0.0	0.0	0.0	12.1	0.0	0.0	19.8	8.3	0.0	30.4	0.0	5.1	0.0	5.1	2.8	21.5	0.0	hsa-mir-6859-4
hsa-miR-877-5p	11.2	24.9	26.7	35.6	0.0	0.0	0.0	14.9	10.2	18.1	0.0	9.6	0.0	0.0	0.0	17.7	0.0	0.0	0.0	11.8	9.4	8.6	3.5	0.0	0.0	0.0	0.0	0.0	0.0	hsa-mir-877
hsa-miR-887-3p	42.2	19.0	34.1	0.0	0.0	48.4	0.0	0.0	0.0	13.0	17.2	0.0	27.5	0.0	0.0	0.0	0.0	0.0	0.0	0.0	4.3	15.3	0.0	14.3	0.0	7.4	9.1	0.0	0.0	hsa-mir-887
hsa-miR-128-1-5p	7.2	17.8	0.0	0.0	0.0	0.0	0.0	0.0	0.0	0.0	12.2	0.0	11.5	6.8	0.0	0.0	58.8	32.3	0.0	0.0	23.9	6.2	5.2	0.0	44.4	3.9	11.9	0.0	8.0	hsa-mir-128-1
hsa-miR-3158-3p	0.0	7.6	9.3	19.1	7.1	0.0	0.0	0.0	0.0	9.8	9.0	36.3	0.0	0.0	17.3	0.0	52.3	14.9	0.0	26.6	0.0	21.1	0.0	0.0	0.0	11.8	0.0	0.0	0.0	hsa-mir-3158-1
hsa-miR-3158-3p	0.0	7.6	9.3	19.1	7.1	0.0	0.0	0.0	0.0	9.8	9.0	36.3	0.0	0.0	17.3	0.0	52.3	14.9	0.0	26.6	0.0	21.1	0.0	0.0	0.0	11.8	0.0	0.0	0.0	hsa-mir-3158-2
hsa-miR-3605-5p	10.8	5.0	20.9	0.0	0.0	0.0	17.2	0.0	0.0	0.0	10.8	0.0	0.0	11.4	0.0	8.8	0.0	0.0	0.0	0.0	0.0	13.4	27.9	0.0	0.0	1.8	0.0	19.3	5.3	hsa-mir-3605
hsa-miR-382-5p	0.0	25.7	8.4	38.2	0.0	0.0	0.0	0.0	0.0	0.0	0.0	0.0	0.0	14.0	0.0	0.0	0.0	0.0	0.0	9.4	20.0	10.3	14.9	0.0	0.0	0.0	10.0	0.0	10.7	hsa-mir-382
hsa-miR-4728-3p	4.6	15.6	0.0	0.0	8.2	16.8	21.4	24.1	9.1	6.2	0.0	0.0	12.5	0.0	15.3	0.0	0.0	0.0	0.0	8.5	0.0	0.0	0.0	24.3	0.0	5.8	20.3	0.0	8.3	hsa-mir-4728
hsa-miR-629-5p	14.0	10.2	12.5	28.5	3.7	0.0	0.0	0.0	0.0	10.1	0.0	0.0	10.8	0.0	0.0	0.0	0.0	0.0	0.0	18.4	29.4	5.8	0.0	0.0	0.0	2.1	5.4	0.0	0.0	hsa-mir-629
hsa-miR-6863	15.7	4.4	9.3	0.0	9.0	0.0	0.0	26.3	0.0	0.0	0.0	14.4	0.0	0.0	0.0	12.4	0.0	0.0	0.0	15.3	0.0	16.3	12.0	11.7	0.0	2.3	0.0	0.0	0.0	hsa-mir-6863
hsa-miR-760	9.4	11.4	0.0	11.2	8.4	0.0	15.3	35.5	0.0	6.8	0.0	19.4	0.0	6.8	14.2	10.0	0.0	0.0	0.0	0.0	5.9	6.9	0.0	0.0	0.0	7.1	4.9	11.3	9.7	hsa-mir-760
hsa-let-7b-3p	5.0	20.0	0.0	0.0	12.5	0.0	19.5	30.3	0.0	9.5	0.0	0.0	25.1	0.0	30.3	6.2	0.0	0.0	0.0	0.0	60.4	9.1	0.0	0.0	0.0	0.0	0.0	0.0	0.0	hsa-let-7b
hsa-miR-100-3p	0.0	13.4	12.2	14.6	0.0	0.0	0.0	28.1	10.8	9.2	0.0	11.8	0.0	18.6	0.0	0.0	0.0	0.0	0.0	0.0	0.0	0.0	0.0	0.0	0.0	3.7	0.0	0.0	0.0	hsa-mir-100
hsa-miR-127-3p	5.0	18.2	30.9	20.2	1.7	4.8	0.0	20.6	2.1	6.8	18.7	5.6	4.2	0.0	0.0	10.9	22.0	3.2	0.4	8.0	6.7	5.8	0.3	0.0	1.0	0.0	0.7	0.0	1.3	hsa-mir-127
hsa-miR-151b	8.6	10.0	9.0	0.0	6.0	14.8	27.1	18.0	0.0	11.3	7.5	0.0	12.2	0.4	0.4	0.0	0.0	0.0	9.1	0.0	0.4	0.0	0.0	0.0	0.0	11.8	7.5	0.0	0.0	hsa-mir-151a
hsa-miR-194-3p	2.2	24.5	20.6	0.0	0.0	16.8	0.0	28.5	0.0	0.0	0.0	0.0	11.5	0.0	0.0	10.9	0.0	0.0	19.4	0.0	0.0	0.2	0.0	0.0	0.0	0.0	0.0	0.0	0.0	hsa-mir-194-2
hsa-miR-20a-5p	4.8	6.4	0.0	0.0	0.0	0.0	0.0	53.1	0.0	0.0	0.0	12.4	0.0	0.0	0.0	44.2	0.0	0.0	0.0	0.0	10.2	19.6	0.0	11.4	0.0	3.5	0.0	0.0	11.0	hsa-mir-20a
hsa-miR-2110	0.0	19.6	18.0	0.0	0.0	8.8	0.0	0.0	9.7	0.0	0.0	0.0	0.0	0.0	18.1	0.0	0.0	0.0	31.4	0.0	10.2	8.1	9.0	20.0	11.4	5.3	6.1	0.0	4.7	hsa-mir-2110
hsa-miR-489-3p	0.0	6.2	0.0	0.0	0.0	0.0	16.4	0.0	0.0	0.0	14.7	0.0	0.0	15.7	18.5	0.0	0.0	0.0	0.0	18.4	0.0	0.0	24.7	9.1	0.0	11.3	0.0	0.0	0.0	hsa-mir-489
hsa-miR-509-3p	32.3	11.6	27.0	0.0	18.7	0.0	0.0	0.0	0.0	0.0	10.4	0.0	0.0	0.0	23.2	12.1	0.0	0.0	0.0	7.8	8.6	0.0	0.0	0.0	0.0	0.0	0.0	0.0	0.0	hsa-mir-509-1
hsa-miR-509-3p	32.3	11.6	27.0	0.0	18.7	0.0	0.0	0.0	0.0	0.0	10.4	0.0	0.0	0.0	23.2	12.1	0.0	0.0	0.0	7.8	8.6	0.0	0.0	0.0	0.0	0.0	0.0	0.0	0.0	hsa-mir-509-2
hsa-miR-509-3p	32.3	11.6	27.0	0.0	18.7	0.0	0.0	0.0	0.0	0.0	10.4	0.0	0.0	0.0	23.2	12.1	0.0	0.0	0.0	7.8	8.6	0.0	0.0	0.0	0.0	0.0	0.0	0.0	0.0	hsa-mir-509-3
hsa-miR-548az-5p	0.0	0.6	28.3	18.0	8.6	0.0	0.4	25.0	8.8	0.0	0.0	13.8	0.0	28.0	0.0	0.0	0.0	0.0	0.0	0.0	10.2	0.0	0.0	0.0	23.9	7.8	7.7	0.0	0.0	hsa-mir-548az
hsa-miR-10a-3p	12.6	11.2	0.0	0.0	0.0	16.5	0.0	0.0	0.0	13.0	0.0	0.0	0.0	0.0	0.4	0.0	0.0	13.2	0.0	7.1	0.0	9.3	0.0	0.0	0.0	3.7	0.0	20.4	0.3	hsa-mir-10a
hsa-miR-125b-1-3p	9.4	6.8	17.0	9.7	6.7	0.0	38.1	0.9	7.0	0.0	0.0	0.0	0.0	0.0	0.0	0.0	0.0	0.0	0.0	7.3	0.0	0.0	10.5	20.5	20.8	3.0	10.0	0.0	1.7	hsa-mir-125b-1
hsa-miR-1269b	0.0	0.0	0.0	0.0	10.1	0.0	0.0	0.0	0.0	0.0	13.3	0.0	0.0	0.0	16.9	9.7	56.2	0.0	0.0	0.0	28.6	0.0	0.0	0.0	0.0	0.0	0.0	15.0	0.0	hsa-mir-1269b
hsa-miR-1304-3p	4.4	9.4	22.5	30.3	0.0	16.0	0.0	0.0	0.0	0.0	0.0	0.0	0.0	0.0	0.0	11.2	0.0	0.0	0.0	10.1	5.5	0.0	0.0	11.7	0.0	1.6	0.0	0.0	2.7	hsa-mir-1304
hsa-miR-193b-5p	22.3	9.4	0.0	15.7	0.0	0.0	0.0	14.9	0.0	0.0	12.6	0.0	0.0	3.4	16.1	0.0	0.0	0.0	0.0	9.0	32.6	0.0	0.0	0.0	0.0	0.0	0.0	0.0	0.0	hsa-mir-193b
hsa-miR-195-3p	13.0	11.2	0.0	0.8	0.0	0.0	0.0	0.0	0.0	0.0	0.0	0.0	10.8	0.0	13.8	0.0	0.0	0.0	0.0	0.0	39.6	9.3	0.0	0.0	0.0	6.2	0.0	0.0	11.0	hsa-mir-195
hsa-miR-23a-3p	10.8	2.6	0.0	0.0	0.2	0.0	0.4	49.1	14.3	0.0	0.0	0.0	0.0	0.0	13.4	0.0	0.0	0.3	15.3	0.2	0.8	0.2	0.3	0.0	0.0	9.9	12.6	0.0	0.0	hsa-mir-23a
hsa-miR-29b-3p	12.8	10.8	10.9	0.0	0.0	0.0	0.0	0.0	0.0	0.0	0.0	0.0	0.0	0.0	0.0	15.3	0.0	0.0	0.0	8.7	0.0	0.0	16.4	0.0	0.0	5.5	24.0	0.0	0.0	hsa-mir-29b-1
hsa-miR-29b-3p	12.8	10.8	10.9	0.0	0.0	0.0	0.0	0.0	0.0	0.0	0.0	0.0	0.0	0.0	0.0	15.3	0.0	0.0	0.0	8.7	0.0	0.0	16.4	0.0	0.0	5.5	24.0	0.0	0.0	hsa-mir-29b-2
hsa-miR-29b-2-5p	5.4	0.0	0.0	0.0	0.0	15.4	0.0	0.0	12.6	0.0	0.0	0.0	0.0	14.8	0.0	0.0	0.0	0.0	0.0	0.0	21.2	19.4	0.3	0.0	23.2	0.0	0.0	0.0	0.0	hsa-mir-29b-2
hsa-miR-301a-3p	5.2	1.8	7.4	14.2	4.1	0.0	0.0	0.0	0.0	7.7	0.0	0.0	0.0	0.0	16.9	18.6	0.0	12.6	0.0	0.0	0.4	0.0	8.0	12.8	0.0	4.2	7.2	0.0	10.3	hsa-mir-301a
hsa-miR-335-3p	9.4	12.8	16.7	0.0	0.2	0.0	0.0	0.0	0.9	9.2	0.0	25.3	8.4	0.0	16.5	0.0	0.0	0.0	0.0	27.3	4.7	0.0	0.0	0.0	6.2	5.8	30.1	0.0	0.0	hsa-mir-335
hsa-miR-34a-5p	10.6	16.2	8.4	0.0	5.6	19.7	22.1	0.0	0.0	0.0	0.0	0.0	0.0	0.0	0.0	12.7	0.0	0.0	0.0	0.0	29.0	0.2	0.0	0.0	0.0	0.0	0.0	0.0	4.3	hsa-mir-34a
hsa-miR-3605-3p	0.0	11.6	0.0	8.6	0.2	0.0	0.0	0.0	0.0	0.0	0.0	0.0	0.0	19.5	33.4	16.2	0.0	0.0	0.0	0.0	1.6	0.0	0.0	2.3	18.0	0.0	10.7	0.0	0.0	hsa-mir-3605
hsa-miR-452-5p	0.0	4.8	11.3	0.0	0.0	0.0	18.3	0.0	0.0	0.0	12.9	0.0	12.5	0.0	0.0	0.0	0.0	0.0	0.0	0.0	0.0	0.0	0.0	0.0	31.9	0.0	9.3	19.3	0.0	hsa-mir-452
hsa-miR-455-5p	0.0	8.2	0.0	0.0	0.0	32.8	0.0	0.0	0.0	9.2	14.4	0.0	0.0	0.0	0.0	13.3	0.0	25.8	0.0	0.0	0.0	9.1	0.0	12.0	27.7	0.0	0.0	0.0	9.0	hsa-mir-455
hsa-miR-664a-5p	8.8	6.8	11.6	0.0	0.0	0.0	21.0	0.0	0.0	0.0	0.0	0.0	0.0	29.2	0.0	0.0	0.0	0.0	0.0	0.0	11.0	7.4	14.2	32.2	0.4	6.5	7.9	0.0	0.0	hsa-mir-664a
hsa-miR-708-5p	0.0	9.8	0.0	21.3	0.0	0.0	22.9	10.1	0.0	0.0	0.0	0.0	9.8	0.0	0.0	0.0	0.0	0.0	0.0	10.4	14.5	8.9	0.0	0.0	23.6	0.0	0.0	0.0	0.0	hsa-mir-708
hsa-miR-769-3p	0.0	9.0	7.4	0.0	0.0	17.1	0.0	0.0	22.8	0.0	0.0	0.0	7.3	0.0	12.2	0.0	0.0	0.0	0.0	7.8	16.1	8.9	11.7	0.0	0.0	5.8	0.0	0.0	18.3	hsa-mir-769
hsa-miR-892a	0.0	11.6	0.0	0.0	17.2	16.8	0.0	0.0	0.0	0.0	0.0	0.0	0.0	0.0	0.0	0.0	0.0	54.2	0.0	0.0	0.0	0.0	0.0	16.0	0.0	5.3	0.0	0.0	11.7	hsa-mir-892a
hsa-miR-106a-5p	4.0	15.8	0.0	0.0	0.0	0.0	0.0	0.0	12.9	0.0	0.0	0.0	0.0	19.5	0.0	13.9	0.0	0.0	0.0	7.3	47.5	0.7	0.3	0.0	0.0	0.2	0.0	0.0	0.0	hsa-mir-106a
hsa-miR-148b-5p	0.0	6.8	0.0	0.0	0.0	3.1	0.0	29.4	0.0	0.0	0.0	0.0	0.0	0.0	17.3	0.0	0.0	14.5	0.0	0.0	0.0	0.0	0.3	11.7	13.2	2.5	0.0	0.0	0.0	hsa-mir-148b
hsa-miR-193b-3p	9.8	6.4	15.8	0.0	0.0	0.0	0.0	0.0	11.4	0.0	16.2	0.0	0.0	0.0	0.0	14.4	0.0	0.0	0.0	0.0	25.5	0.0	0.0	0.0	0.0	5.1	0.0	0.0	0.0	hsa-mir-193b
hsa-miR-3065-3p	10.0	10.0	4.2	0.0	0.0	0.0	0.0	0.0	11.7	0.0	0.0	0.0	0.0	15.7	0.0	15.3	0.0	0.0	21.1	9.2	7.5	0.0	0.0	0.0	0.0	0.5	0.0	0.0	0.0	hsa-mir-3065
hsa-miR-330-5p	0.0	0.0	0.0	0.0	0.0	17.1	0.0	0.0	0.0	21.0	0.0	10.7	0.0	0.0	0.0	0.0	0.0	0.0	0.0	8.5	0.0	0.0	12.5	0.0	13.2	0.0	4.9	0.0	0.0	hsa-mir-330
hsa-miR-582-3p	0.0	10.4	9.3	37.4	9.2	0.0	0.0	0.0	0.0	10.1	4.7	0.0	0.0	0.0	0.0	0.0	0.0	0.0	0.0	9.9	10.2	28.5	0.0	0.0	0.0	0.0	0.0	0.0	0.0	hsa-mir-582
hsa-miR-625-3p	0.0	5.4	14.8	0.0	0.0	17.9	0.0	0.0	9.7	0.0	13.3	0.0	0.0	0.0	0.0	0.0	0.0	0.0	0.0	8.0	3.9	6.5	0.0	0.0	0.0	10.4	12.6	0.0	0.0	hsa-mir-625
hsa-miR-939-5p	22.3	4.0	0.0	0.0	0.0	0.0	28.2	0.0	0.0	13.0	10.8	0.0	0.0	0.0	0.0	0.0	0.0	5.2	0.0	3.5	5.1	16.3	0.0	0.0	0.0	2.5	0.0	0.0	0.0	hsa-mir-939

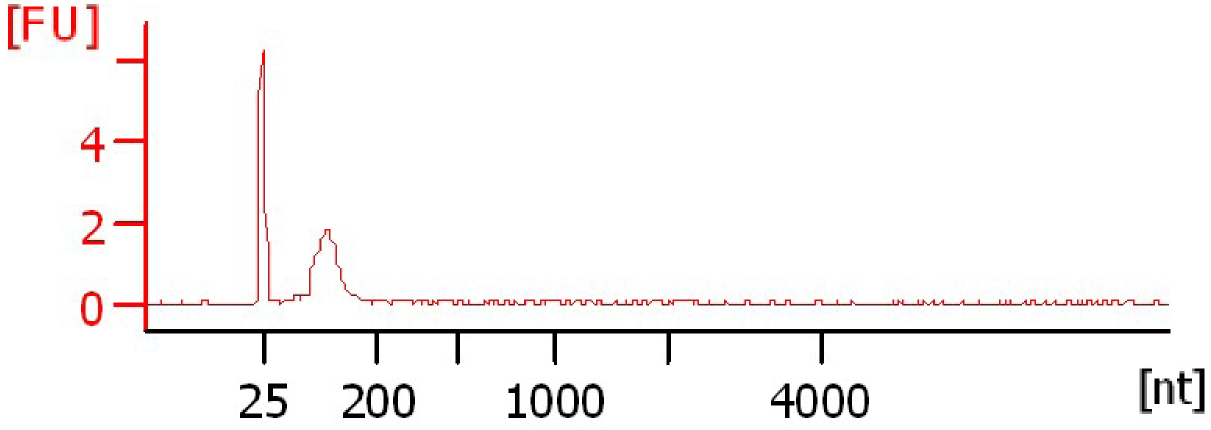

- Figure S1 -

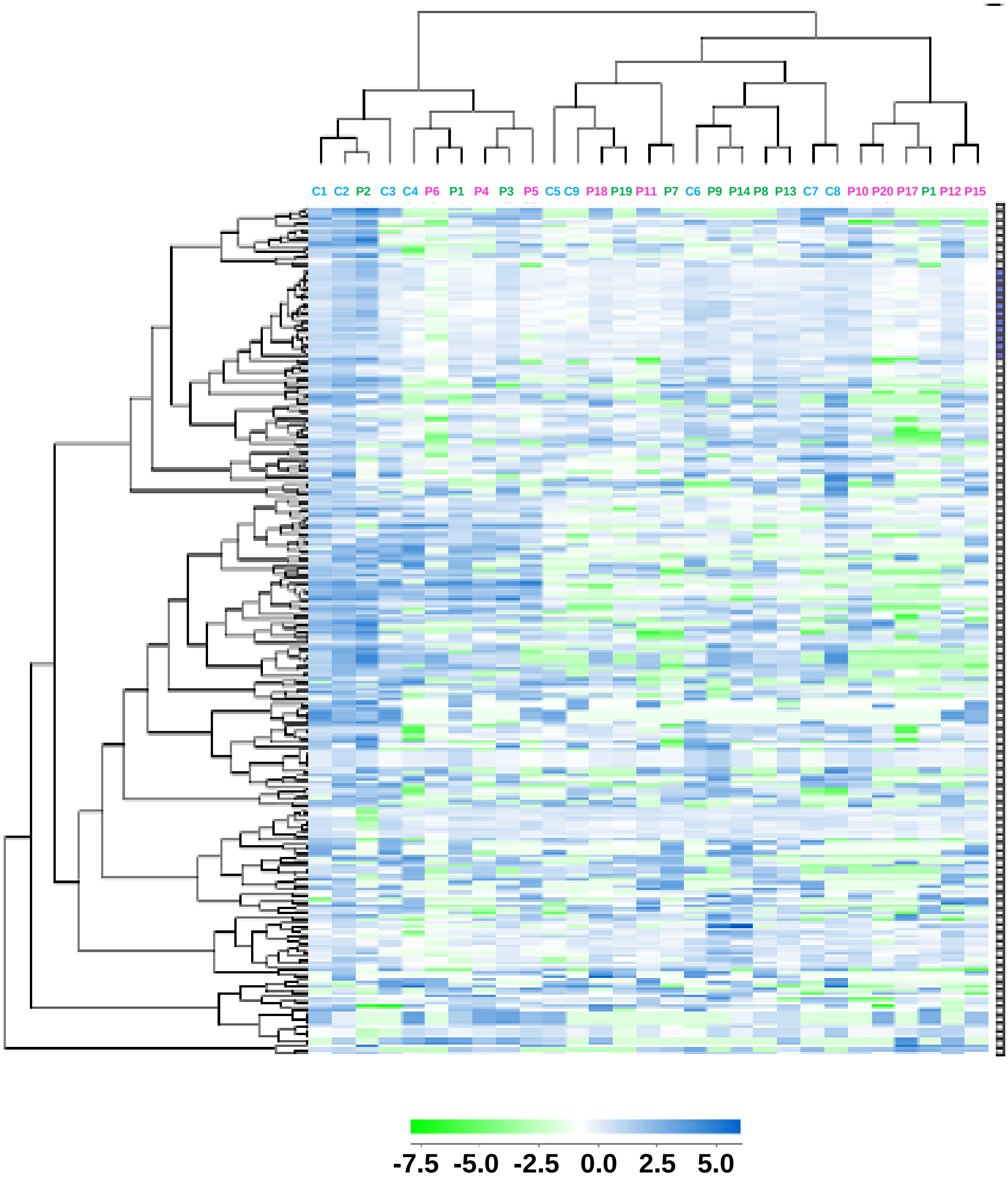
C1 C2 P2 C3 C4 P6 P1 P4 P3 P5 C5 C9 P18 P19 P11 P7 C6 P9 P14 P8 P13 C7 C8 P10 P20 P17 P1 P12 P15
-7.5 -5.0 -2.5 0.0 2.5 5.0

# - Figure S2 -

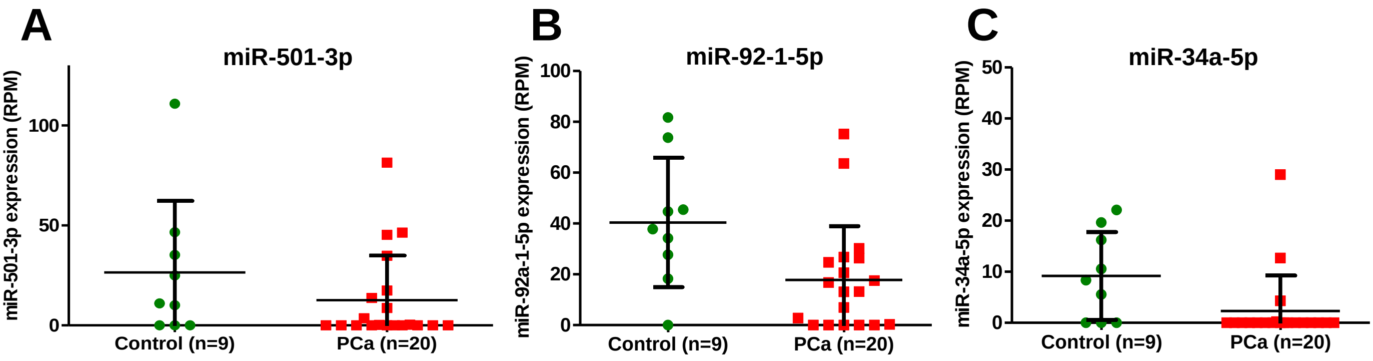
A
miR-501-3p
miR-501-3p expression (RPM)
100
50
0
Control (n=9)
PCa (n=20)
B
miR-92-1-5p
miR-92a-1-5p expression (RPM)
100
80
60
40
20
0
Control (n=9)
PCa (n=20)
C
miR-34a-5p
miR-34a-5p expression (RPM)
50
40
30
20
10
0
Control (n=9)
PCa (n=20)

# - Figure S3 -

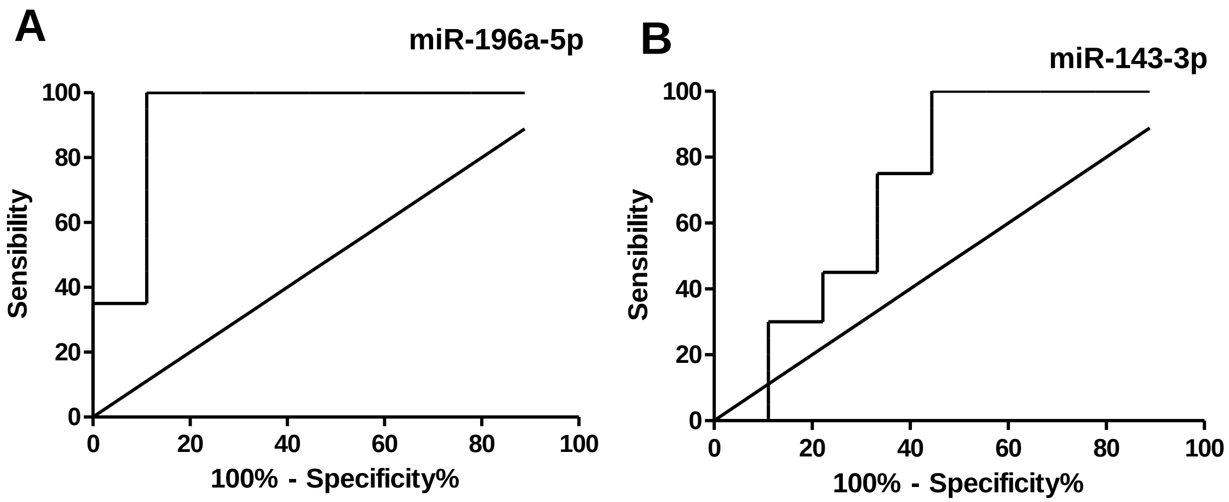
A
miR-196a-5p
Sensibility
100% - Specificity%
B
miR-143-3p
Sensibility
100% - Specificity%

- Figure S4 -

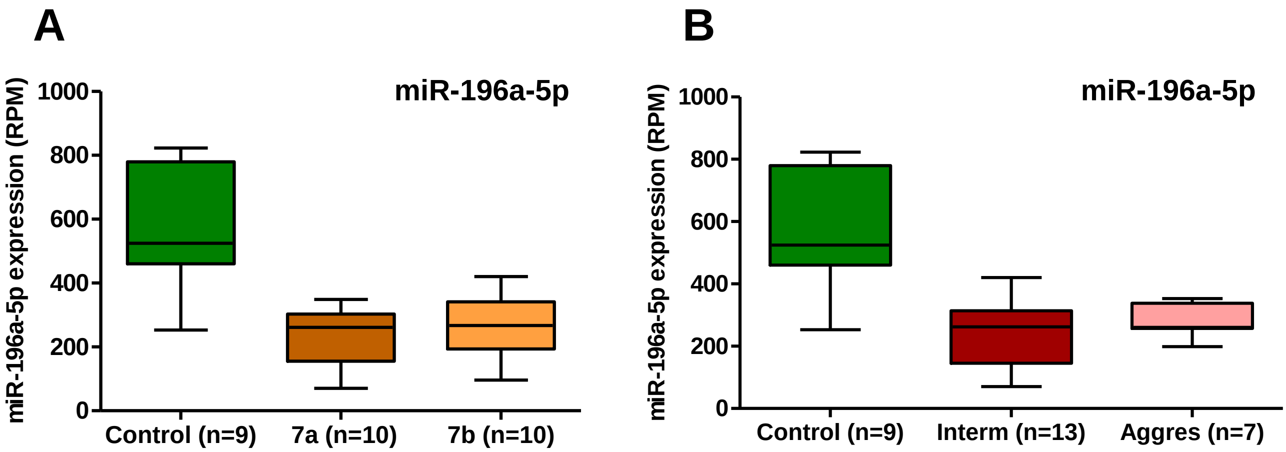
A
miR-196a-5p
miR-196a-5p expression (RPM)
1000
800
600
400
200
0
Control (n=9)
7a (n=10)
7b (n=10)
B
miR-196a-5p
miR-196a-5p expression (RPM)
1000
800
600
400
200
0
Control (n=9)
Interm (n=13)
Aggres (n=7)

- Figure S5 -

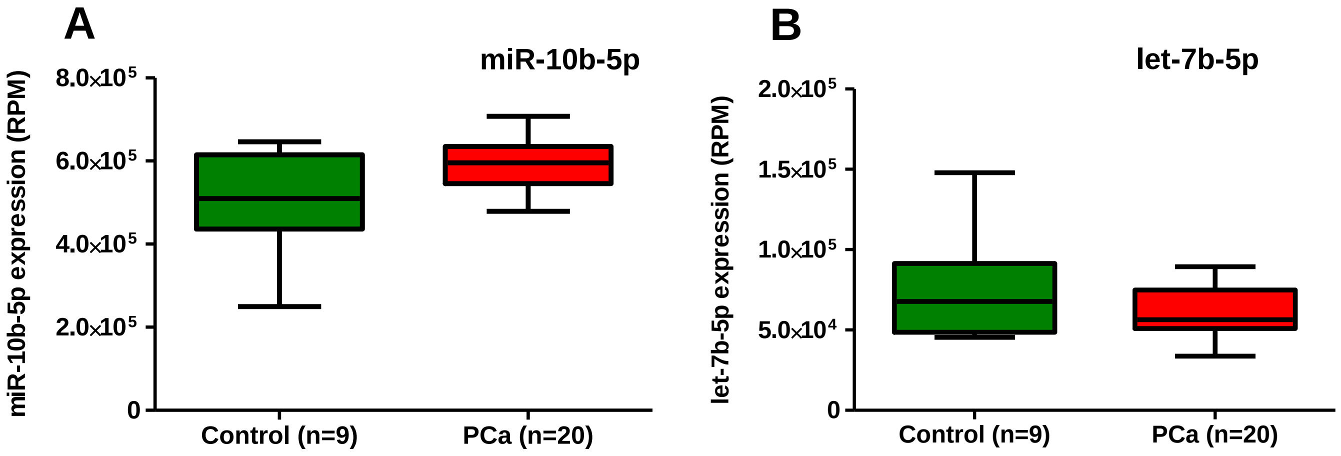
A
miR-10b-5p
miR-10b-5p expression (RPM)
8.0×10⁵
6.0×10⁵
4.0×10⁵
2.0×10⁵
0
Control (n=9)
PCa (n=20)
B
let-7b-5p
let-7b-5p expression (RPM)
2.0×10⁵
1.5×10⁵
1.0×10⁵
5.0×10⁴
0
Control (n=9)
PCa (n=20)

- Figure S6 -

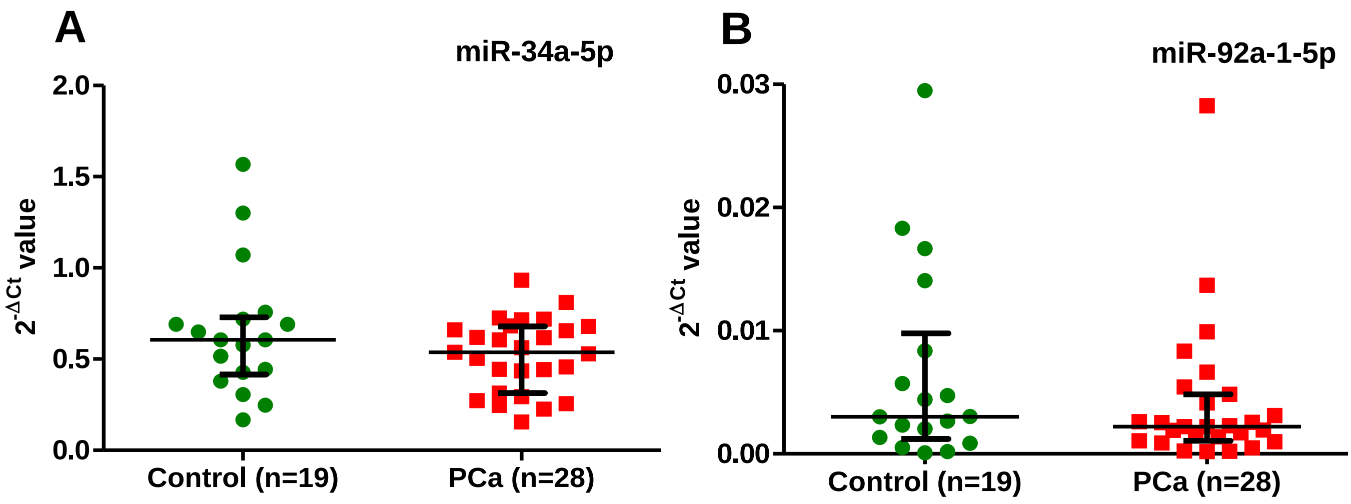
A
miR-34a-5p
2.0
1.5
1.0
0.5
0.0
$2^{-\Delta Ct}$ value
Control (n=19)
PCa (n=28)
B
miR-92a-1-5p
0.03
0.02
0.01
0.00
$2^{-\Delta Ct}$ value
Control (n=19)
PCa (n=28)

- Figure S7 -

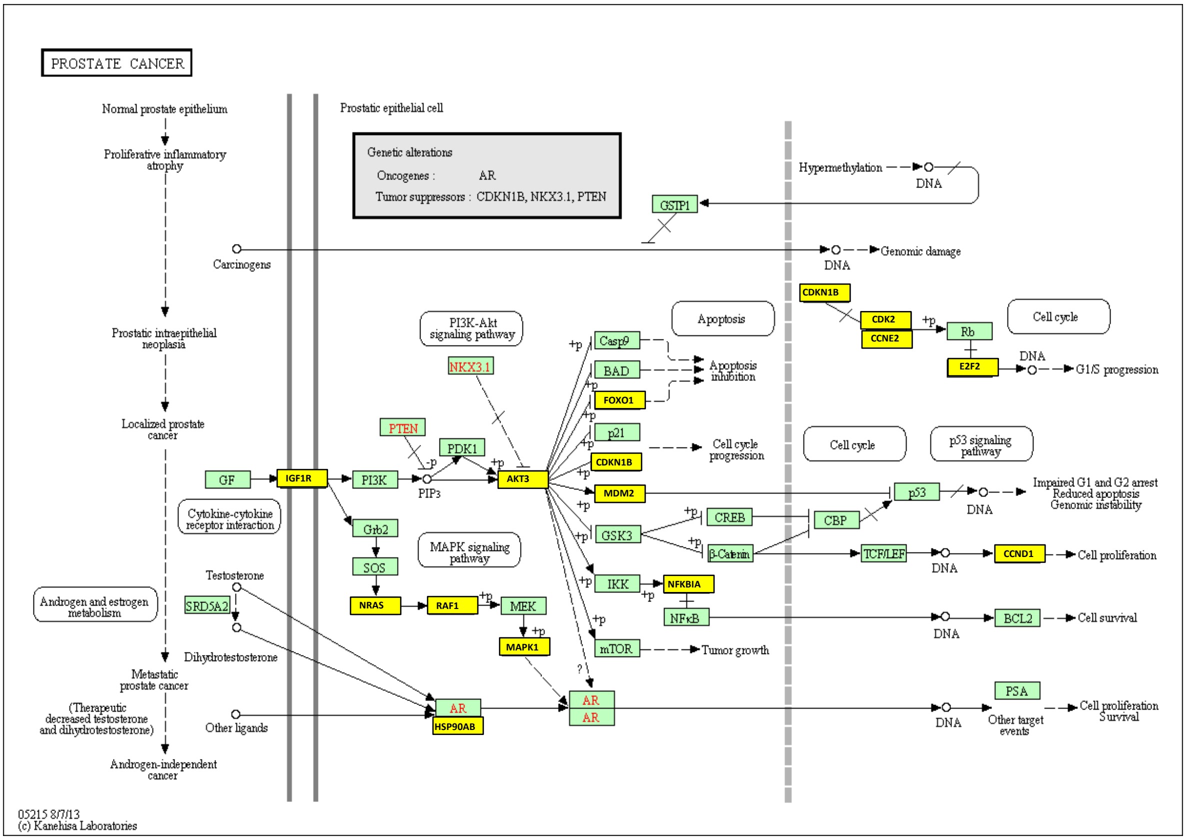

PROSTATE CANCER
Normal prostate epithelium
Proliferative inflammatory atrophy
Prostatic intraepithelial neoplasia
Localized prostate cancer
Metastatic prostate cancer
(Therapeutic decreased testosterone and dihydrotestosterone)
Androgen-independent cancer
Prostatic epithelial cell
Genetic alterations
Oncogenes : AR
Tumor suppressors : CDKN1B, NKX3.1, PTEN
Hypermethylation
DNA
GSTP1
Carcinogens
Genomic damage
CDKN1B
CDK2
CCNE2
Rb
E2F2
Cell cycle
G1/S progression
Apoptosis
Apoptosis inhibition
PI3K-Akt signaling pathway
NKX3.1
PTEN
PDK1
Casp9
BAD
FOXO1
p21
CDKN1B
Cell cycle progression
p53 signaling pathway
GF
IGF1R
PI3K
PIP3
AKT3
MDM2
p53
Impaired G1 and G2 arrest
Reduced apoptosis
Genomic instability
Cytokine-cytokine receptor interaction
Grb2
SOS
MAPK signaling pathway
GSK3
CREB
CBP
β-Catenin
TCF/LEF
CCND1
Cell proliferation
Testosterone
SRD5A2
Androgen and estrogen metabolism
NRAS
RAF1
MEK
MAPK1
IKK
NFKBIA
NFκB
BCL2
Cell survival
mTOR
Tumor growth
Dihydrotestosterone
AR
HSP90AB
Other ligands
PSA
Other target events
Cell proliferation
Survival
05215 8/7/13
(c) Kanehisa Laboratories

- Figure S8 -
